# Aptamer-Nanoconjugates as Potential Theranostics in Major Neuro-Oncological and Neurodegenerative Disorders

**DOI:** 10.3390/pharmaceutics17091106

**Published:** 2025-08-25

**Authors:** Roxana-Georgiana Tauser, Florentina-Geanina Lupascu, Bianca-Stefania Profire, Andreea-Teodora Iacob, Ioana-Mirela Vasincu, Maria Apotrosoaei, Oana-Maria Chirliu, Dan Lupascu, Lenuta Profire

**Affiliations:** 1 Department of Pharmaceutical and Therapeutical Chemistry, Faculty of Pharmacy, University of Medicine and Pharmacy «Grigore T. Popa» Iasi, 16 Universitatii Street, 700115 Iasi, Romania; roxana.tauser@umfiasi.ro (R.-G.T.); andreea.panzariu@umfiasi.ro (A.-T.I.); ioana-mirela.vasincu@umfiasi.ro (I.-M.V.); apotrosoaei.maria@umfiasi.ro (M.A.); oana-maria.ionescu@umfiasi.ro (O.-M.C.); dan.lupascu@umfiasi.ro (D.L.); lenuta.profire@umfiasi.ro (L.P.); 2 Department of Internal Medicine, Faculty of Medicine, University of Medicine and Pharmacy «Grigore T. Popa» Iasi, 16 Universitatii Street, 700115 Iasi, Romania; bianca-stefania.profire@umfiasi.ro

**Keywords:** aptamer-nanoconjugates, glioma, amyloidopathies, aptasensors, aptamer-chimeras, targeted drug delivery

## Abstract

This review aims to point out the main achievements in the cutting-edge field of aptamer nanotechnology and its applications in the most frequent neuro-oncological and neurodegenerative diseases. The article discusses the properties, advantages and drawbacks of aptamers (AP), and their design and selection by various SELEX methods, as well as the synergical advantages as theranostics of the aptamer-functionalized nanoparticles (Ap-NP). The Ap-nanoconjugates properties are compared to those of Ap and unconjugated NP. Moreover, the article comparatively analyzes the aptamer-based approaches vs. antibody-drug conjugates vs. exosome-based delivery systems vs. unconjugated NP, as targeted therapies in neurodegenerative diseases and gliomas. The review presents major challenges in Ap-NP conjugates’ clinical progress (concerning the in vivo enzymatic stability, blood–brain barrier (BBB) permeability, selective intracellular uptake in the brain parenchyma and target tissues, rapid renal clearance, off-target toxicity, immunogenicity, reproductible manufacturing) and the investigated developmental strategies to solve them. Furthermore, relevant examples and comparative insights regarding preclinically tested Ap and Ap-NP conjugates are presented for targeted delivery systems loaded with chemotherapeutical drugs or genes, Ap-siRNA chimeras and immunotherapeutical aptamers, which are evaluated in glioblastomas (GBM), amyloidogenic diseases and multiple sclerosis (MS); radiotherapy enhancers in GBM; aptasensors for diagnostic and bioimaging-guided therapy in GBM, MS and amyloidopathies. The review finally points out future research directions in order to accelerate the clinical translation and the real-world impact as theranostics of the most preclinically advanced Ap-NP conjugates in major neuro-oncological and neurodegenerative disorders.

## 1. Introduction

Aptamers (Ap), etymologically derived from the Latin “aptus” (to fit) and the Greek ‘‘meros’’ (particle), are integrated within nucleic acid-based and oligonucleotide (OGN) pharmaceuticals, alongside nucleic acid vaccines, antisense oligonucleotides, immunomodulatory DNA/RNA, gene therapy, small interfering RNA (siRNAs), microRNA (miRNAs), circular RNA (circRNAs), plasmids and gene-editing guide RNA (gRNA). To date, 21 oligonucleotide-based pharmaceuticals are approved in the USA and the EU (of which 12 are antisense OGN; 2 aptamers; 6 siRNAs, single-stranded and double-stranded combined polydeoxyribonucleotides) and hundreds are at different preclinical and clinical testing [1,2]. Aptamers are single-stranded oligonucleotides (10–100 nucleotides) with three-dimensional 3D conformations endowed with high selectivity and binding affinity to a huge diversity of targets whose minimum size is at least 60 Daltons (small molecules, metal ions, peptides, proteins, 3D complex molecular biomolecules, bacteria, viruses, yeast, mammalian cells, tissue, animals) [3,4]. If other OGN therapies’ essential role resides on regulation of a particular gene’s transcription or translation, aptamers act primarily by their 3D conformations. Aptamer-based theranostics, aptasensors and vectors for targeting drug delivery are gaining increasing interest in the field of precision medicine and pharmaceutical research. In 2004, the FDA approved the first therapeutical aptamer pegaptanib sodium (Macugen), an RNA-modified aptamer conjugated to polyethylene glycol (PEG), aimed for intravitreal injection, acting as antagonist of vascular endothelial growth factor (VEGF) indicated for the treatment of age-related macular degeneration [5,6]. Recently, in August 2023, avacincaptad pegol (Izervay) also received FDA approval for therapy of the geographic atrophy secondary to age-related macular degeneration [7]. Intense research is equally dedicated to aptamers as therapeutical agents, diagnostic biomarkers and brain drug-targeting delivery systems in precision medicine regarding brain cancers, neurodegenerative diseases and other neurological disturbances [8,9].

Glioblastoma multiforme (GBM) is a high-grade glioma characterized by the highest incidence, malignancy and fatality rate; short median survival (12–15 months, after maximal surgical ablation and chemoradiation therapy; 9 months in pediatric patients with diffuse midline glioma carriers of *H3K27M* mutation); high relapse rates; increased therapeutic resistance to chemo- and radiotherapy of the residual tumor cells due to heterogenicity and complexity of tumor microenvironment (TME); it is also incurable among brain tumors [10,11].

In precision medicine, the design of tailored GBM-targeted aptamers focuses on specific antiproliferative aptamers targeting biomarkers of GBM cell surface or TME; aptamers as delivery systems across blood–brain barrier (BBB) of antineoplastic drugs; aptamers as conjugates with functionalized nanoparticles loaded with anticancer drugs; aptamers-conjugates with small interfering RNA as gene silencing’s mediators [12,13].

Neurodegenerative diseases (NDs) are caused by formation of neurotoxic oligomers, accumulation and deposition of misfolded and post-translationally aberrantly-modified proteins, as well as of the aggregates derived from microtubule associated protein Tau. These could be illustrated by hyperphosphorilated neurofibrillary tangles, in tauopathies like Alzheimer’s disease (AD), frontotemporal lobar degeneration, progressive supranuclear palsy, and chronic traumatic encephalopathy. Synucleinopathies, such as Parkinson’s disease (PD), dementia with Lewy bodies and multiple system atrophy, are caused by brain deposits of aggregated neurotoxic oligomers derived from synuclein [14,15,16]. Prionoses (i.e., Creutzfeldt–Jakob disease, Gerstmann–Sträussler–Scheinker syndrome, fatal familial insomnia) are due to deposition of neurotoxic aggregates of the misfolded and oligomerized prionic infectious proteins PrPSc (scrapie form) with typical cross-β structure of amyloid fibrils, which are highly stable and resistant to enzymatic proteolysis and denaturation, and which derive from the cellular prionic form PrPC. PrPC is involved in neuroprotection and trophic signaling, but also in binding amyloid β Aβ oligomers which are responsible for triggering neuronal dystrophy and synaptotoxicity under genetic, environmental, or yet unknown factors [17,18]. Therefore, amyloidogenic proteins, including Aβ, Tau, αSyn, and PrPSc represent potential therapeutic targets for specific anti-amyloid aptamers. For example, Aβ42, the 42-amino acid residue form of amyloid fibrils in senile plaques in the AD, aggregates faster into higher molecular-weight oligomers which are more neurotoxic than Aβ40; moreover, both Aβ42 and Aβ40 oligomers are more neurotoxic than the corresponding fibrils; all these previously mentioned amyloid aggregates could be targets for aptamers [19].

The purpose of this review is to underline the great potential of aptamers and aptamers’ conjugates with nanoparticles, which are currently preclinically and clinically tested in gliomas, tauopathies and other neurodegenerative diseases. The review will progressively discuss the following issues: aptamer properties, advantages and drawbacks; the synergical advantages as theranostics of the aptamer-functionalized nanoparticles; the investigated developmental strategies to solve major challenges in Ap-NP conjugates’ clinical development. Ap-nanoconjugate properties are also compared to those of Ap and NP alone. Moreover, comparative analysis is also made on the advantages and limitations of the aptamer-based approaches vs. antibody-drug conjugates vs. exosome-based delivery systems vs. unconjugated NP, as targeted therapies in neurodegenerative diseases and gliomas. Relevant examples, comparative analysis and critical insights regarding preclinically tested Ap and Ap-NP conjugates are discussed for CNS-targeted delivery systems loaded with chemotherapeutical drugs or genes, Ap-siRNA chimeras, aptabody, which are evaluated in gliomas, amyloidogenic diseases and multiple sclerosis (MS), as radiotherapy enhancers in GBM, as well as aptasensors for diagnostic and bioimaging-guided therapy in GBM, MS and amyloidopathies. The review also points out future research directions in order to accelerate the clinical translation and the real-world impact as theranostics of the most preclinically advanced Ap-NP conjugates in major neuro-oncological and neurodegenerative disorders.

## 2. Aptamer Properties

### 2.1. Aptamer Classification, Advantages and Drawbacks

According to their structure, aptamers are classified into nucleic acid and peptide aptamers. Nucleic acid (NA) aptamers are short (20–100 base pairs) single-stranded OGN derived from structures of either DNA or RNA (ssDNA or ssRNA), which fold into 3D conformations exclusive to NA, such as hairpins, internal loops, bulges, triplicates, stem-loop complexes, crosses, pseudoknots, and G-quadruplexes. RNA-based aptamers have higher affinity for targets due to their superior conformational stability, which is explained by the presence of the hydroxyl group in ribose (not in deoxyribose) and the absence of methyl group in uracil (not in thymine) [16]. Moreover, RNA aptamers are more structurally flexible than DNA aptamers, but RNA-based aptamers have lower chemical and enzymatic stability and they require more processing steps. Peptide aptamers are conformationally restricted loops of polypeptides (5–20 residues) due to lower enthropic energy, which might enable them even 1000 times higher binding affinity than the corresponding free peptides [20,21].

Aptamers bind to their biological targets by favorable van der Waals forces, electrostatic interactions, hydrogen bonding, stacking of flat moieties, and 3D shape complementarity [16].

Aptamers have unique and advantageous biophysical and biochemical properties, such as small size and great flexibility to adopt 3D foldings and conformations; customizable design to achieve remarkable pleiotropy; specificity and binding affinity to a wide diversity of targets; low or even non-immunogenicity; increased enzymatic stability and favorable blood clearance through versatile chemical modulations; facile computerized in silico design and scalable synthesis (either enzymatical, by solid-phase chemical synthesis or in silico) with low production costs; and potential to develop as ligands for brain drug delivery targeting [22,23].

Aptamers are considered true chemical antibodies (mAb) or antibody-mimic materials, with which they share the highly specific interactions to the targets (similar to antibodies, aptamers could detect targets in the micro- to picomolar range) and binding affinity in nanomolar range of equilibrium dissociation constants [24].

However, aptamers lack the main disadvantages of mAb, such as immunogenicity, high prices, instability at room temperature, poor batch reproducibility, and impermeability through an intact BBB [10]. For instance, the molecular weight of typical aptamers is approximately 5–10 times smaller (~12–30 kDa; ~30–80 nucleotides on average) than typical IgG antibodies (150–170 kDa), and even at doses 1000 times higher they lack toxicity and immunogenic risk associated with mAb [25]. Aptamers have remarkable stability at room temperature for prolonged storage periods and they do not require special handling conditions; moreover, their denaturation is reversible. In contrast to therapeutic mAb, chemically modulated DNA and RNA aptamers can be stored for long periods of time at low temperature or as lyophilized products, they are readily reconstituted, and can support repeated freeze–thaw cycles [26]. In addition, chemical synthesis of aptamers through the SELEX process uses nucleic acid sequences with great structural diversity, and in small quantities of a few nanomoles; it also facilitates the addition of various functional moieties and enables fast (~1-3 months) and cost-effective production, with a high degree of batch-to-batch reproducibility, without contamination risk and the need for cell cultures or animals as required for mAb. Moreover, aptamers could be also designed for unidentified heterogeneous targets from various tissues and cells [4,27].

Furthermore, an expanded comparative analysis of the advantages offered by the aptamers in comparison to other emerging technologies, such as antibody-drug conjugates (ADC) or exosome-based delivery systems, is illustrated in Table 1.

**Table 1:** Comparative analysis of aptamer-based approaches vs. antibody-drug conjugates vs. exosome-based delivery systems in neurodegenerative diseases and gliomas.

Aspect	Aptamer-Based Approaches	Antibody-Drug Conjugates (ADC)	Exosome-Based Delivery Systems	Refs.
Targeting specificity	high	very high	natural (via exosome surface proteins)	[8,19,27,28,29,30,31,32,33,34]
Size and BBB penetration	small (~15–30 kDa) excellent BBB penetration	5–10× larger (~150 kDa) limited BBB penetration without modification	small vesicles (30–150 nm); naturally cross BBB efficiently	
Stability in circulation	enhanced by chemical modulations	stable	stable; protects cargo from degradation	
Immunogenicity	low	moderate-high	low	
Cargo delivery	efficient and versatile (drugs, siRNA, miRNA etc.)	chemotherapeutics; limited nucleic acid delivery	versatile (proteins, RNA, drugs; can be engineered for enhanced loading)	
Manufacturing cost	low, cost-effective	expensive	moderate (purification challenging)	
Clinical progress	mostly preclinical in brain diseases; in clinical trials for cancers (e.g., Ap AS1411); Ap-conjugates in clinical trials in brain diseases	multiple FDA-approved ADCs in oncology (e.g., Brentuximab vedotin, Polatuzumab vedotin); limited trials in gliomas	several early-phase clinical trials for exosome therapeutics ongoing (not yet in neurodegenerative diseases)	

The main drawbacks of chemically unmodified aptamers are related to an altered pharmacokinetic profile due to high sensitivity to serum nuclease-mediated degradation (the unmodified aptamers usually have a half-life T1/2 in the order of minutes), to DNAses family 1 predominant in extracellular fluid and to RNAases present in serum and cerebrospinal fluid (CSF); pH-dependent stability, especially RNA aptamers which are hydrolyzed at pH 5.5; rapid distribution to tissues from the plasma; and quick clearance by rapid renal filtration. In addition, other barriers towards Ap’s clinical developmental derive from unintended cross-binding to structurally-related targets (off-target toxicity), potential immune reactions and manufacturing scalability [35,36,37]. These major challenges and investigated solutions to solve them are discussed further in this review.

### 2.2. Aptamer Design and Selection

**Figure 1:**
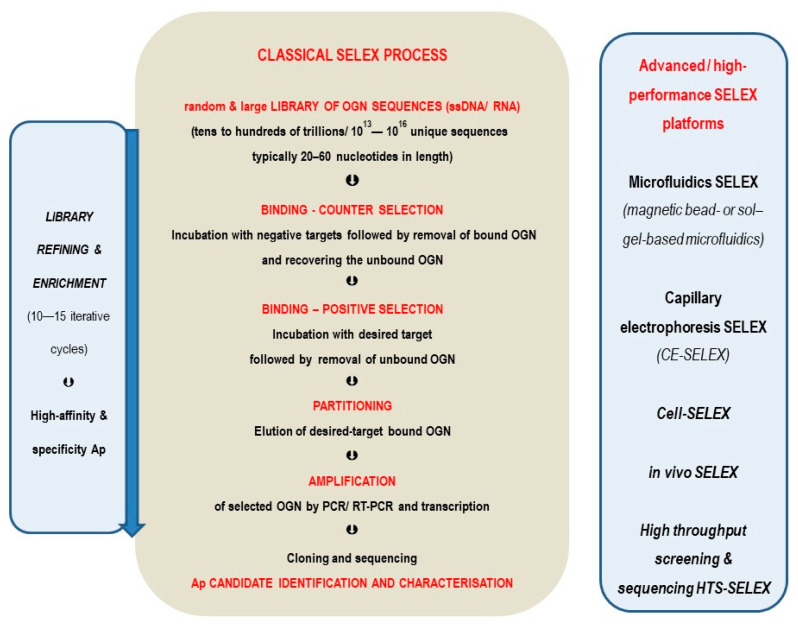
Classical steps of SELEX process and examples of advanced SELEX platforms.

High-affinity DNA- and RNA-aptamers are synthesized using the systematic evolution of ligands by exponential enrichment (SELEX) technique from large libraries (tens to hundreds of trillions) of randomly synthesized OGN, typically 20–60 nucleotides in length, specific to purified proteins or small molecules. The classical SELEX steps are as follows: (1) counter selection step, when the primary library (10^13^–10^16^ ssDNA/RNA) is incubated with undesired, negative targets, followed by removal of bound OGN and recovering the unbound OGN; (2) positive binding, i.e., further incubation with desired specific purified target followed by removal of unbound OGN; (3) partitioning step, i.e., elution of desired target-bound OGN; (4) amplification of selected OGN by polymerase chain reaction (PCR)/Real-Time PCR (RT-PCR) and transcription; (5) cloning and sequencing for Ap candidate identification and characterization. The SELEX technique usually applies 10–15 iterative cycles of steps (1) ⇨ (5) for library refining and enrichment. These steps are schematically illustrated in Figure 1 and described in detail elsewhere [10,38,39].

Neomer library, a recent aptamer collection with 16 random nucleotides intertwined with fixed sequences, enables identification of aptamers with binding affinity to unknown plasma biomarkers predictive for Alzheimer’s (AD) and other neurodegenerative diseases, with specificity of 0.76 and sensitivity of 0.88. Neomer juxtaposes its starting collection against multiple plasma probes, without the pre-enrichment phase from conventional methods [38].

After aptamer selection on SELEX, the solid phase synthesis of aptamer-based medicines is highly dependent on the length of OGN sequence and is challenged by the necessity of high yields, scalability costs, and long manufacturing periods, while preserving product purity and synthesis accuracy [38,39].

Further developments of the SELEX technique into higher performance ones are also depicted on the right side of the Figure 1 as (a) microfluidics SELEX (magnetic bead- or sol–gel-based microfluidics), which uses custom-designed devices and enables minimum reagent consumption, automated control, multiplexing/on-chip/high-throughput detection and screening, and increases the efficiency of incubation and separation steps and reduces off-target bindings; (b) capillary electrophoresis SELEX (CE-SELEX) which proved very efficient and quick (2–4 selection cycles, within days instead of weeks) for isolation of high-affinity aptamers; (c) cell-SELEX enabling aptamer identification for many living mammalian cells types without deciphering the exact structure of the molecular target and without the protein purification step; (d) in vivo SELEX—aimed for aptamers exposed to the physiological complexity of whole animals—in which the aptamer library is injected into a living animal and after a certain time the aptamers are recovered from the target tissue or sub-cellular compartment (brain, tumors, etc.) [39,40,41].

These advanced technology-driven SELEX strategies isolate aptamers within a few days and comprise high-throughput screening (HTS) based on next-generation sequencing (HTS-SELEX). Computational methods used for in silico aptamer identification, design and optimization are created using molecular docking, molecular dynamics simulation, quantitative structure–activity relationship, hybrid quantum mechanics/molecular mechanics, and machine learning-based methods and algorithms based on sequence-, motif-searching- and multi-dimensional- scoring [10,42]. For example, Murakami et al. [20] describe the following steps for in silico aptamer design and optimization: aptamer candidates are clustered by HTS analysis programs (i.e., meta-Z-score, AptaCluster); the clusters’ secondary structures and motifs are predicted by software like MFold, MEMERIS, and/or QGRS Mapper; aptamers’ tertiary structures are generated by tools like RNAComposer, VMD, SimRNA or other algorithms available at Rtools [43]; the prediction of G-quadruplexes in RNA and DNA sequences is made with QGRS (Quadruplex forming G-Rich Sequences) Mapper, GRSdb, 3DNus or QuadBase2; molecular docking of the aptamers with their target protein enables the identification of the binding site(s) by machine learning tools (i.e., FTDock, GROMACS); aptamers’ affinity and/or specificity is further tested and improved by SMARTAptamer and AptaMut analysis; the consequences of point mutations on binding affinity and chemical and enzymatic stability are simulated by AptaMut; and optimization of the aptamer geometry is performed with GAMESS, FMO [1,20,41,44]. Moreover, “SOMAScan” is a commercial aptamer-based proteomics tool that enables the identification of potential biomarkers of mental diseases as targets for aptamers by screening in one sample of CSF over 7,000 different proteins against specific aptamers [10]. Furthermore, aptamer-based SOMAScan^®^ proteomic assay technology has enabled the identification of 24 serum proteins linked to CD133, which are involved in neuro-oncology signaling pathways, with great potential as clinically valuable predictive biomarkers for diagnostics, treatment monitoring and 1-year survival prediction in GBM [45,46].

## 3. Aptamer-Nanoparticles (Ap-NP) Conjugates

### 3.1. Aptamer-Functionalized Nanoparticles: Synergical Advantages as Theranostics

As a cutting-edge intersection domain, aptamer nanotechnology focuses on engineered aptamer-conjugated nanohybrids as innovative theranostics, enabling increasing benefits in targeted drug delivery, controllable drug release and in vivo bioimaging-guided therapy. Ap-NP can encapsulate a variety of medicines and payloads, such as chemotherapeutics, proteins, genes, siRNAs, radionuclides, lipids, metals etc. Ap-nanoconjugates are already designed as drug delivery systems (cargo delivery; carrier role), therapeutic candidates, biosensors (aptasensors) and bioimaging-agents (surface imaging, in vivo intracellular molecular imaging) [47,48].

Ap-NP conjugates are composed of various nanomaterials, like nucleic acids, gold/silver/carbon-NP, magnetic, quantum dots, polymers, dendrimers, and hydrogels. Nanomaterials are characterized by large surface areas (especially for nanosheets, nanoflowers), unique quantum properties, great biocompatibility and versatile functionalization possibilities, thus enabling multiple cargo delivery and target interactions. Nanoparticles excel as nanocarriers with great drug delivery efficiency (high payload and controlled release), but need targeting moieties to avoid off-target accumulation and immunotoxicity [49,50].

Among the great variety of NP classified upon dimensions, structure and composition, organic NP (like liposomes, dendrimers, polymers) are very biocompatible and preferred in bioimaging and drug transportation and delivery; inorganic NP (gold, silver, iron oxide, carbon NP) and liposomes can penetrate deeply into tissues; liposomes as drug carriers also enable prolonged half-time; nanoclusters have reduced in vivo toxicity. Gold NPs make the best conjugates with aptamers, have large surface areas and surface plasmon resonance and allow versatile chemical modulations [48,51,52]. Nucleic acid (DNA or RNA) NPs have water solubility, base pairing and self-assembling abilities, as well as high potential for drug–aptamer conjugation; protein-mimic NP can bind with high affinity to mRNA and efficiently deliver it intracellularly, thus enabling gene expression modulation [36,53,54]. Polymeric, silica or gel NPs also serve as protective matrix for the encapsulated Ap, thus effectively increasing the enzymatic stability and targeted delivery of Ap-NP conjugates [55].

Aptamer-functionalized nanoparticles synergistically combine advantages of aptamers’ targeting ability with nanoparticles’ customizable delivery. Thus, Ap-NP conjugates have improved stability, prolonged half-life, targeting precision due to increased binding affinity and specificity, biocompatibility, and large surface areas, as well as versatility of secondary and tertiary conformational modulations. However, they introduce manufacturing complexity and greater safety evaluation requirements before clinical use. A comparative analysis among Ap, NP and Ap-NP conjugates regarding aspects, such as targeting specificity and affinity, size and biodistribution, stability, immunogenicity and toxicity, drug-loading capacity, synthesis and manufacturing, and clinical translation status, is summarized in Table 2 [2,25,29,55,56,57,58,59,60].

### 3.2. Ap-NP Conjugates vs. ADC/mAb-NP vs. NP as CNS-Targeted Therapies

As CNS-targeted therapies in GBM and neurodegenerative diseases, Ap-NP conjugates have proven robust preclinical results and currently offer the strongest combination of specificity, high payload versatility and delivery, enhanced BBB penetration, low immunogenicity, modular targeting capacity and practical scalability in preclinical research in comparison to ADCs, mAb-NPs and NPs alone [30,61,62].

In these pathological contexts, ADCs are still emerging and non-targeted NPs lag behind. ADCs remain constrained by payload limits, large size, and production complexity, though mAb-functionalized nanoparticles partially alleviate these drawbacks. Moreover, ADCs/mAb-NPs are significantly challenged by antibody stability, immunogenicity, manufacturing cost, and off-target toxicity after linker degradation. Limitations of NPs encompass poor CNS targeting by passive delivery, limiting their therapeutic efficacy and increasing off-target accumulation; therefore, they serve better as general carriers than targeted therapeutics [4,10,11,27].

Table 2 also presents a comparative analysis of various properties of Ap-NP, ADC/mAb-NP and NP regarding various features: BBB penetration and targeting precision; drug loading and delivery capacity; specificity and affinity; versatility (customization and modularity, size); safety and immunogenicity; manufacturing scalability; stability in circulation; clinical relevance; and translational challenges. Aptamer-NPs and mAb-NPs provide active, receptor-targeted BBB crossing (e.g., transferrin receptor TfR targeting), with aptamer systems offering customizable, small-target modularity, while simple NPs rely on passive mechanisms and are typically less precise and less effective without ligand functionalization. ADCs face substantial delivery challenges to the CNS due to limited BBB permeability, although strategies like antibody-functionalized nanoparticles could mitigate this by high drug payload capacity and leveraging receptor-mediated uptake [49,53,57,59].

Concerning drug loading capacity and multivalency, as well as delivery capacity, ADCs typically carry 2–4 drug molecules per antibody (drug–antibody ratio), while mAb–NP hybrids can load ~10× more payload due to NP’s core. Ap-NP conjugates exhibit similar high multivalency, delivering siRNAs, small molecules, or photosensitizers (mesoporous NP) in glioma models, thus achieving significant therapeutic impact. Nanoparticle-based conjugates (Ap or mAb) enable higher payloads than ADCs, increasing therapeutic efficacy per targeting moiety [63,64].

Comparing the specificity, affinity and versatility of the Ap-NP conjugates, with those of ADC/mAb-NP and unconjugated NP, respectively, Ap functionalization enables rapid and flexible engineering of bispecific conjugates, due to smaller sizes than mAb and minimum steric hindrance. On the contrary, Ab are rigid and expensive, especially for bispecific targeting, while bare NPs lack targeting specificity unless functionalized. Moreover, Ap-functionalized NPs allow rapid design iterations and multiplexing (e.g., bifunctional aptamers binding TfR and Tau) [4,10,11,62].

Aptamers and Ap conjugates shine in low immunogenicity and reduced off-target toxicity, and ADCs have narrow therapeutic windows with toxicological risks from cleavage products, while NPs alone are characterized by undesired accumulation in various organs and oxidative stress associated to metal-NP [57,59].

Although Ap-NP conjugates are synthetically scalable, their clinical translation is slowed by inconsistent production, variability in batch quality, and stability issues. ADCs are biologics requiring complex biomanufacturing. NPs (lipid or polymeric) benefit from industrial-scale methods (e.g., high-pressure homogenization), improving scalability, but require precise ligand attachment and conjugation control [4,6,11,27,30,49,53,57,62].

**Table 2:** Comparative analysis of various properties of Ap-NP conjugates, ADC/mAb-NP and NP.

Property	Ap-NP Conjugates	ADCs/mAb–NP Hybrids	Aptamers	NP Alone	Ref.
Targeting precision & Binding specificity and affinity	High combines Ap specificity with NP multivalency; Ap can be engineered for disease-specific biomarkers	High strong affinity but rigid in design; specific Ab difficult to re-engineer	Very high modifiable for recognizing any target	Intrinsically low, no active targeting; enhanced by ligand functionalization	[2,4,6,10,11,25,27,29,30,49,53,55,56,57,58,59,60,62,63,64]
Payload capacity & Drug versatility	High multiple drugs/small molecules, siRNA, genes, imaging agent molecules per NP	Moderate limited to ~2–4 drugs per antibody; chemotherapeutics primarily	Medium	High and broad—depends on carrier type: drugs, siRNA, contrast agents etc.	
Delivery control	High synergistic delivery: specific targeting + loading capacity; stimuli-responsive release possible	Moderate relies on linker cleavage	High	Passive, often burst release	
Size and biodistribution	Higher (~50–200 nm); deep tissue penetration superior biodistribution	Larger antibodies reduced tissue penetration	12–30 kDa (30–80 nucleotides in average); fast renal clearance	10–200 nm; size-dependent biodistribution; can exploit permeability and retention effect (EPR)	
Stability in circulation	Moderate—increased by Ap-functionalization or surface chemical modulations (PEGylation etc.)	High IgG-based Ab have long T1/2	Sensitivity to nucleases without chemical modulations	Variable depends on coating material (organic, nucleic acids, inorganic)	
BBB penetration	High receptor-mediated transcytosis (e.g., TfR, Tau); dual-targeting systems (like TfR–Tau circular aptamer)	Limited for native ADCs Moderate for mAb-NPs	Customizable active BBB crossing	Low mostly passive, size-dependent	
Immunogenicity and toxicity	Low (non-proteinaceous); Ap reduces immunotoxicity; depends on NP core	Moderate protein-based—risk of immune reactions	Low immunogenicity manageable	Low (without surface conjugation); toxicity varies on composition (liposomes/polymers safe; metals may be toxic)	
Customization and modularity	Excellent rapid and low-cost SELEX modifications	Limited engineering is complex and costly	High chemical modulations	Requires surface modification	
Manufacturing and scalability	High complex chemical synthesis (linker chemistry, conjugation control, purification); scalable production	Low biologics specific manufacturing	Chemically synthesized; scalable	High established NP methods; lipid NPs industrially produced	
Limitations	Nuclease degradation, regulatory complexity	Immunogenicity, high cost; BBB remains a major barrier	Moderate drug loading capacity	Lack of targeting; off-target effects; poor CNS specificity	
Clinical translational outlook	Promising translation but not yet approved; most preclinical—strong efficacy in gliomas and tumour brain imaging; few in ongoing clinical trials	Moderate—ADCs approved for cancer; early progress in brain disorders as BBB remains a major barrier	Limited (Pegaptanib, Avacincaptad approved); most preclinical	Several approved (e.g., Doxil, Onpattro)	

### 3.3. Obtaining Ap-NP Conjugates

Ap-NP conjugates have been obtained either by covalent or non-covalent attachment of aptamers to NP, or by their encapsulation within a protective matrix [65,66]. The most cited methods are covalent conjugations (directly by amide bonds or through a linker), followed by non-covalent interactions mediated either by the highly stable biotin-streptavidin moiety or by linkers with reciprocal electrical polarity.

Covalent conjugations are more common and stable than non-covalent interactions, because they ensure enhanced structural stability and integrity, as well as prolonged in vivo functionality of the Ap-NP bioconjugate. Covalent bonds could be thioester, amide, ester, disulfide ones, among the thiol, carboxylic or aldehyde functional groups present on aptamer and nanomaterials, respectively. These covalent interactions can be either directly formed, or by intercalating a linker between the aptamer and nanomaterials which has a critical role in flexibility. For instance, PEG or maleimide polyethyleneglycol are linkers intercalated between the sgc8 DNA aptamer and the liposome surfaces. Besides the functional groups involved, the efficient covalent conjugation also depends on optimal reaction parameters (pH, temperature, solvent) in order to ensure good yields, stability and functionality of both components [36,37,67].

Non-covalent conjugations reside on hydrogen, van der Waals, ion–ion electrostatic interactions, and *π-π* stacking, either directly or through a linker; for instance, in indirect conjugation, a negatively charged aptamer interacts with a positively-charged linker and the latter is immobilized to the negatively-charged surface of NP [55,60].

## 4. Ap-Nanoconjugate Developmental Strategies

### 4.1. Chemical Modulation Strategies Addressing In Vivo Stability, Target Binding Affinity and Renal Clearance

Chemical modification strategies aim mainly to improve aptamers’ half-life (up to several days), stability to nucleases, cellular uptake, renal clearance, and BBB permeability. These chemical modulations may be applied to each structural motif (nitrogenous base, phosphodiester backbone, deoxyribose/ribose sugar) either after the aptamer’s isolation, or during the selection step of already chemically modulated library, with the essential requirement of the compatibility with enzymatic polymerase chain reaction [40,60]. The main chemical modulations and their beneficial consequences are illustrated in Table 3. For instance, 5′- or 3′-terminal PEG- or *N*-acetylgalactosamine modulations induce resistance to rapid human glomerular filtration and increase intracellular uptake, respectively, while phosphorothioate or phosphorodiothioate backbones, as well as locked nucleic acids as substitutes for sugars, render much more resistance to nucleases. Modified hydrophobic nucleotides at three internal and two terminal positions, such as hydrophobic 3,5-bis(trifluoromethyl)benzoyl analogs, are favorable to increasing base pairing interactions and affinity, tissue-specificity, association with serum albumin, systemic delivery and in vivo stability [10,68].

**Table 3:** Improvement of Ap properties through various chemical modulations.

Improved Properties	Applied Chemical Modulations on Structural Motif of			Ref.
	Nitrogenous Base	Phosphodiester Backbone	Deoxyribose/Ribose Sugar	
Stability to nuclease degradation	hydrophobic nucleotides at 3 internal and 2 terminal positions (e.g., hydrophobic 3,5-bis(trifluoromethyl)benzoyl analogs)	phosphorothioate (PS); phosphorodithioate (PS2) replacement	2′-deoxy-2′-fluoro-d-ara- binonucleic acid (2′-F ANA); locked nucleic acid (LNA); unlocked nucleic acid (UNA)	[10,22,38,39,40,60,69,70]
		methylphosphonate or triazole substitution	2′-fluoro, 2′-*O*-methyl and 2′-amino-substitutions	
	chiral inversion: Spiegelmers (*L*-enantiomeric oligonucleotide aptamers)		2′–OCH3 and 20-*O*-ribose alterations (20-*O*-methyl/20-aminopyrimidine-/20-fluoro-/20-deoxypyrimidines)	
			biotin-streptavidin conjugation to 3′-end	
			3′-end capping with inverted (deoxy-)thymidine (idT); 3′-3′ or 5′-5′ inversion	
Resistance to renal clearance	-	-	PEG or cholesterol or dialkyl lipids attached to 5′-end	[7,10,38,39,60,68,71]
	-	-	biotin-streptavidin conjugation to 3′-end	
			PEG- or *N*-acetylgalactosamine attached to 5′- or 3′-end	
Increasing binding affinity and specificity for target	5-(*N*-benzylcarboxyamide)-2-deoxyuridine, SOMAmers (Slow Off-rate Modified Aptamers)			[22,38,39,41,60,69,70]
	5-(*N*-naphtyl/-triptamino/-isobutyl-)carboxyamide-2-deoxy uridine SOMAmers			
	hydrophobic nucleotides at 3 internal and 2 terminal positions, such as hydrophobic 3,5-bis(trifluoromethyl)benzoyl analogs			

Spiegelmers are *L*-RNA aptamers also created by the SELEX process which have enzymatic stability due to their non-natural stereoisomeric configuration and also very low immunogenicity. Spiegelmers like NOX-A12 (olaptesed pegol), NOX-E36, NOX-H94, are already investigated in clinical trials (see further in this review). For instance, synthetic *L*-nucleotides were used by NOXXON Pharma for Spiegelmers aptamers endowed with stability to serum nucleases, low immunogenic potential and anti-tumor activity (neutralization of chemokines within TME), being now in clinical trials [70].

### 4.2. Investigated Strategies to Tackle Major Challenges in Ap-NP Conjugates’ Clinical Development

Moving forward, clinical development of Ap-NP conjugates as successful neuro-oncological and neurodegenerative medicines is still confronted by major challenges concerning enzymatic stability, BBB permeability, rapid renal clearance, long-term biocompatibility, immunotoxicity, manufacturing scaling-up, and regulatory hurdles.

**Figure 2:**
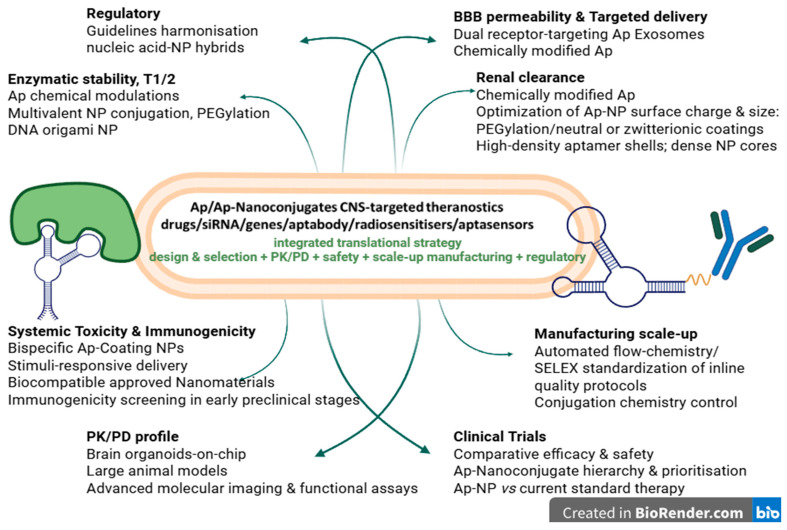
Investigated strategies to solve major challenges in Ap-NP conjugates’ clinical development.

Recent strategies and technological innovations investigated in order to solve these challenges combine chemical Ap modulations and stabilization, multivalent NP conjugation, receptor-specific selection, and innovative delivery systems. Chemical modulations of Ap (already detailed in Table 3) and integration of Ap within DNA nanostructures have enabled enhanced enzymatic stability, controlled release and reduced off-target effects [10,38,39,40,41,57]. The following paragraphs describe other strategies researched to address these major challenges and they are depicted in Figure 2.

1. Enzymatic stability, T1/2

Multivalent NP conjugation using dense aptamer shells on NPs creates steric hindrance and charge repulsion, reducing enzymes’ access and stabilizing Ap-NP conjugates in serum. Moreover, high-molecular weight nanoparticles as carriers might further slow renal filtration, prolonging blood retention of Ap-NP. PEGylated branched polymers on NP surfaces have allowed ultralong circulation time (e.g., carbon nanotube T1/2 ≈ 22 h). In addition, PEGylation of *L*-aptamers exceeds renal filtration thresholds. Incorporation of Ap into DNA origami nanostructures has enhanced the stability, controlled release, and reduced off-target effects [38,39,68].

2. BBB permeability

Dual receptor-targeting aptamers, exosome-mediated aptamer delivery to the brain, adjunct BBB disruption technologies, as well as implementation of Microphysiological System–SELEX (MPS SELEX) in Ap selection, are among the most investigated solutions to BBB penetration. Bifunctional Ap-NP systems using dual receptor-targeting aptamers—e.g., TfR/epithelial cell adhesion molecule (EpCAM) and Tau—can selectively cross the BBB and have demonstrated enhanced in vivo brain uptake of the therapeutic cargo (e.g., doxorubicin or anti-amyloid agents). Brain cell-derived exosomes loaded with ATP-responsive DNA aptamers leverage endogenous BBB transport, achieving efficient in vivo brain delivery for bioimaging agents. As adjunct BBB disruption technologies, focused ultrasound combined with microbubbles have transiently permeated the BBB and boosted brain delivery of NPs. Moreover, hBS01 aptamer, identified by human BBB organ-on-chip SELEX, has shown strong brain accumulation in vivo due to clathrin-mediated transcytosis [8,13,14,55,72,73,74].

3. Rapid renal clearance

The most investigated strategies refer to optimization of surface charge and hydrodynamic size, PEGylation or neutral/zwitterionic coatings, conjugation to poly(lactico-glycolic) acid (PLGA), chitosan, and nanoparticles with dense or high-density cores. Increasing effective hydrodynamic size (>~5–6 nm) of Ap-NPs is correlated to reduced renal glomerular filtration. High-density aptamer shells reject enzymatic degradation and hinder renal excretion. Ap PEGylation increases hydrodynamic radius, reduces reticuloendothelial system (RES) uptake, delays renal clearance (especially of metal-NPs; ~40% metal NPs being excreted in 24 h) and also avoids off-target accumulation of larger NPs in the liver/spleen/brain. Conjugation to PLGA or chitosan also extends in vivo circulation to 4–28 h. Dense (e.g., gold) or high-density cores can increase resistance to rapid renal elimination better than low-density counterparts (silica), enhancing circulation retention. Moreover, gold nanoclusters protected by glutathione have shown both high tumor uptake and a sophisticated balance between retention and renal elimination [14,19,31,75,76].

4. Systemic toxicity

Surface conjugation with dual-targeted aptamers enhances active targeting, reduces reticuloendothelial clearance, and improves accumulation at disease sites, thus reducing systemic toxicity. Dual-targeting strategies using bispecific aptamers or aptamer cocktails enhances specificity by requiring simultaneous binding, reducing off-target interactions and systemic toxicity. Stimuli-responsive engineered NPs to release cargo only in TME conditions (pH, enzymes) might also limit systemic exposure [6,26,30,46].

Cytotoxicity of metal-NP is diminished by Ap-conjugation. For example, at 100–200 µg/mL serum concentrations, Ap-coated metal-NPs have demonstrated negligible cytotoxicity, unlike unmodified controls, and even after repeated injections, they did not alter in vivo liver and renal functional and histological markers. In addition, coating Ap-nanoparticles with PEG and neutral ligands significantly diminishes cytotoxicity.

Oxidative stress is mainly associated to metal-core NP (Fe, Au, ZnO) which can catalyze reactive oxygen species generation and ion release. Ap coatings or chelating coatings applied on metal-NPs shield metal ions and diminish the risk of oxidative chain reactions, at greater tolerated doses. In addition, exosome covering and biodegradable materials improve clearance and safety profiles. Moreover, targeted and stimuli-responsive designs minimize off-target effects and systemic toxicity [55,72,73,75,77].

5. Immunotoxicity

Immunotoxicity might be mediated by CpG motifs, which can activate innate immune responses; surface negative charges, which might bind to off-target proteins and activate Toll-like receptor–mediated immune responses. Coating NPs with Ap shells reduces NP’s opsonization and aggregation, and improves solubility. Biocompatible carriers (like exosomes) and PEGylation might also reduce immune activation. Surface PEGylation and zwitterionic polymer coatings on Ap-nanoconjugates mask immunogenic epitopes, reduce protein corona formation and improve circulation stability. Anti-inflammatory agents or immunomodulator co-delivery strategies might also diminish local and systemic inflammation and immune reactions. In addition, implementing immunogenicity screening in the early stages of preclinical testing would greatly contribute to a faster translational success [8,11,53,55,69,74].

6. Long-term biocompatibility

This aspect could be improved by using FDA-approved nanoparticle materials which are biocompatible and biodegradable (e.g., PGLA, lipids, exosomes, PEG), thus reducing long-term accumulation and toxicity. Exosomes and extracellular vesicles as natural delivery of NPs avoid recognition by mononuclear phagocyte system and inflammatory response, prolonging circulation time and lowering toxicity, in comparison to synthetic NPs [22,35,74,77].

7. Manufacturing scaling-up

Reproducible and large scale manufacturing is challenged by Ap-NP’s surface conjugation complexity, limitations of currently obtaining procedures, batch-to-batch reproducibility, cost and yield, and regulatory hurdles. For instance, improper, uneven orientation or overcrowding of Ap on the surface of Ap-NP conjugates might block binding sites after SELEX selection. Tight control over conjugation chemistry is critical to ensure batch-to-batch reproducibility. Some techniques, like co-extrusion, microfluidic templating, and spray drying, are hard to translate from lab to manufacturing without compromising consistency and sterility. Variability in NP’s physicochemical traits (size, zeta potential, drug loading, Ap density) also complicates good manufacturing practice (GMP). Current high synthesis costs for some modified aptamers and PEGylated nanopolymers, as well as low yields of small-scale SELEX pipelines, can also interfere with scaling up of Ap-NP conjugates [8,13,14,75,78].

In addition, Ap-NP conjugates are multi-component systems (nucleic acids + nanocarrier + drug) requiring complex regulatory pathways for assessing purity, sterility, biostability. Automated flow-chemistry/SELEX, modular click-conjugation and a more rigorous control over conjugation chemistry, might contribute to better batch-to-batch reproducibility. Furthermore, optimization and standardization of the quality protocols, including inline quality control, should be addressed [55,73,79].

8. Regulatory

Ap-NP conjugates are complex multicomponent theranostics systems which require an adequate regulatory profile concerning both nucleic acid therapeutics and nanomedicines and covering a comprehensive assessment of all the above described issues (1–7). The current lack of standardized guidelines for multifunctional conjugates slows clinical translation. Early dialogue between manufacturing companies and regulatory institutions, standardization initiatives and harmonization of complex multicomponent quality standards would ensure faster translation of preclinical advances towards clinical success [6,8,30,46,64,69].

### 4.3. Ap-NP Conjugates’ Targeted Delivery Across the BBB

From neuro-oncological and neurodegenerative perspectives, aptamers’ targeted delivery across the BBB into brain parenchyma or tumors remains one of the major challenges, which is tackled by their encapsulation into natural exosomes or synthetic nanoliposomes with conveniently manipulated surface biomarkers. In order to alleviate the immunogenic potential of exosomes, it is preferred to use exosomes extracted from the patient-derived cells, although it is more time-consuming and expensive [80]. On the other hand, nanoliposomes ensure some advantages over exosomes as brain targeted delivery tools for aptamers, such as greater synthetical versatility regarding their composition and cargo, and easier and cheaper large scale production with higher yields [8,80].

For instance, aptamer-exosome conjugates able to cross the BBB and tested for neurodegenerative diseases could be illustrated by aptamers F5R1, LJM-3064, sgc8 and hBS01. DNA aptamer F5R1 binds to fibrillary alpha-synuclein αSyn and was encapsulated in rabies virus glycoprotein-coated exosomes; after intraperitoneal administration in a murine model of synucleinopathy, this F5R1-exosome conjugate avoided passing through the BBB because it was retrogradely transported by trans-synaptic transmission through peripheral neurons towards central nervous system, where it diminished αSyn aggregation in the substantia nigra and also motor disturbances in mice [20,26]. LJM-3064 aptamer-exosome conjugate is targeted to myelin, stimulating remyelination and reducing the severity of inflammation in a C57BL/6 murine model of multiple sclerosis [32]. sgc8-Aptamer-functionalized exosomes were obtained by conjugation of the aptamer sgc8 to diacyllipid through a PEG linker and afterwards by attaching it to exosomes derived from immature dendritic cells. These sgc8-aptamer-functionalized exosomes specifically deliver docetaxel (DTX) into CCRF-CEM cancer cells [20]. Aptamer hBS01 acted as brain-targeting delivery system of high protein payloads across the human BBB by clathrin-mediated endocytosis; thus, hBS01 efficiently increased the in vitro and in vivo accumulation of the high protein payloads within brain cells [80,81,82].

Aptamer-drug conjugates have proven more cost-effective, lower immunogenic and a simpler alternative to even bispecific (bivalent) mAb as targeted vectors for drugs. Targeted drug delivery systems use functionalized aptamers and they are designed by different approaches, such as intercalating an aptamer, a linker and a drug, or bivalent aptamers specific for different biomarkers bearing as a linker a double-stranded dsDNA loaded with drug, or aptamer-antibody conjugates, or as aptamer-functionalized nanoparticles [83].

For instance, branched aptamer-drug conjugates composed of various OGN sequences, enzymatically-cleavable linkers and payloads, can be illustrated by the system composed of the 46 DNA bases aptamer targeting the GR-20 region of the overexpressed epidermal growth factor receptor (EGFR) in brain tumors, the payload monomethylauristatin E, the cathepsin-cleavable ValCit-*p*-aminobenzylcarbamate linker and the branching situs pentaerythritol-based tetraazide, that can encapsulate different drugs [84]. Furthermore, aptamer-bound DNA nanoflowers, integrating aptamer sequences precisely into the rolling ring amplification templates of self-assembled DNA, serve as brain-drug delivery vectors and theranostics [85].

Another solution for the improvement of the permeability across the BBB, internalization to target cells and binding dynamics speed, might reside in the truncation of aptamers longer than 50 nucleotides by excising nonfunctional domains from OGN sequence [7]. For example, Gli-35 is a truncated aptamer developed from longer sequence Gli-55 aptamer by molecular modeling techniques of hairpin conformational and structural optimization; this technique allows lower manufacturing costs and rapid identification of the critical OGN sequences for binding which are present in Gli-55. On the contrary to Gli-55, Gli-35 have shown increased selective binding to glioblastoma cells (Gli-55 binds also to glial fibrillary acidic protein), fixation dynamics and GBM uptake, thus it could be a better imagistic and GBM drug delivery system [8].

### 4.4. Ap-NP Conjugates as Targeted Delivery Systems for Drugs/Genes

The aptamer role resides on precisely targeting the NP loaded with classical drugs or gene medicines to particular tissue or cell biomarkers, thus increasing the selectivity and the efficiency of the incorporated drug’s delivery and minimizing unwanted effects on other cells. After the aptamer-NP conjugate’s endocytosis into the target cells, the drug payloads will be released from the NPs in response to various stimuli or intracellular factors [86,87].

Aptamer-nanocarrier conjugates have been designed with DNA/RNA origami, nanoliposomes, polymeric micelles, gold nanoparticles, carbon nanotubes, silicon nanomaterials. Polymeric NPs as nanocarriers loaded with drugs and having aptamer-modulated surfaces offer great perspectives on improvement of various features, like low immunogenicity; biocompatibility and biodegradability; increased binding selectivity and sensitivity; large drug-loading ability; expanded circulation time; increased stability to nucleases; avoidance of rapid renal filtration; improved drug efficacy; controllable drug intracellular uptake and release; diminished undesired cytotoxicity; enhanced permeability and retention (EPR) effect into tumoral tissues; as well as significantly enhanced efficiency as targeted drug delivery systems to specific pathological tissues, tumors and inflammation sites [88,89]. Furthermore, they can be produced cost-effectively in bulk quantities and functionalized with diverse chemical modifications, thus offering a versatile design as drug delivery systems (i.e., stimuli-, adenosine triphosphate concentration ATP-, near-infrared or pH-responsive drug release) or as real-time imaging agents [22,55].

For instance, aptamer-functionalized nanoparticles loaded with docetaxel (DTX) showed enhanced anti-cancer effects and cellular uptake of DTX [75,89]. Aptamer-polypro drug conjugate is characterized by augmented in vivo stability to nucleases; controlled drug release kinetics; prolonged in situ recirculation period; and increased cytotoxicity to tumors [73,76].

Selective drug delivery was obtained by aptamer conjugation to dendrimers and hyperbranched polymeric drug nanocarriers, such as A10 RNA aptamer specific to prostate specific membrane antigen (PSMA) coupled by a PEG linker to dendrimer polyamidoamine and loaded with miR-15a and miR-16-1 drugs in order to target LNCaP cancer cells, and has significantly suppressed the expression of *BCL2*, *WNT3A*, *CCND1* genes; dibenzocyclooctyne-modified sgc8 aptamer is conjugated with poly(ethyleneglycol)acrylate and with photo-responsive hyperbranched polymer loaded with DTX, thus achieving increased hydrophilicity and half-time, intratumoral permeability, as well as controllable and very selective cytotoxicity against CCRF-CEM cancer cells (a type of human leukemic lymphoblasts commonly used in immune-oncology) [56,90,91].

Aptamer-coated micelle nanocarriers have also the advantages of selective targeting, superior intracellular delivery of drugs and decreased off-target, systemic toxicity. Multivalent aptamers targeting tumor-specific biomarkers (i.e., nucleolin, human epidermal growth factor receptor EGFR2, mucin-1 MUC1, protein tyrosine kinase PTK7 etc.) can be attached on micelle nanocarriers by base-pairing hybridization, click chemistry or conjugation mediated by 1-ethyl-3-(3-dimethylaminopropyl)carbodiimide [92]. For instance, aptamer AS1411 increased the cellular uptake of doxorubicin (DOX) into nucleolin-positive MCF-7 cancer cells through nucleolin-mediated endocytosis; thus, it enables higher anti-cancer efficacy and lower cardiotoxicity of DOX in mice bearing MCF-7 tumors than administration of the free DOX [69,93].

Ap-NPs can act also as vectors for gene therapy. Anti-EpCAM (epithelial cell adhesion molecule) or anti-VEGF aptamers coated with polyamidoamine and conjugated with mesoporous silica nanoparticles proved potent anti-angiogenic effects due to inhibition of in vivo and in vitro EGFR expression and increased the tumor-targeted efficiency of the encapsulated CRISPR and Cas9 plasmid therapy that is regarded as “magic scissors” [82,94,95].

## 5. Preclinical Aptamers-Nanoconjugates in Glioblastoma and Other Brain Tumors

### 5.1. Preclinical Ap-NP as Targeted Delivery Systems Loaded with Chemotherapeutical Drugs

The main Ap-nanoconjugates as targeted delivery systems for chemotherapeutical drugs which are preclinically tested are illustrated in Table 4, pointing out their major findings and current limitations.

The 26 bases-oligonucleotide AS1411 aptamer has a G-quadruplex structure and binds with high affinity to nucleolin (NCL) overexpressed on tumor cells. AS1411 downregulates the astrocytes’ exosome-miRNA-27a-mediated cross-activation of glioblastoma by the following sequential events: binding to NCL, translocation into nucleus and binding to NF-κB pathway transcription factor P65, downregulation of miRNA-27a and upregulation of the target gene of miRNA-27a in gliomas (INPP4B), and finally the inhibition of the PI3K/AKT pathway and of glioma proliferation. Among the preclinically tested aptamers, AS1411 is the most used and has advanced into phase I and II clinical trials. In other cancer clinical studies, it shows significant and persistent anti-tumoral effects after 6–9 months post-therapy, reduced toxicity, usually at intravenous (i.v.) doses of 1 mg/kg/day [10,47,73,76]. Relevant nanoconjugates based on *AS1411* aptamer (Table 4) comprise the following: AS1411–PGG–PTX; AS1411–DOX Nanosphere; AS1411–NP loaded with PTX; as well as dual aptamers Tetrahedral DNA tFNA (GS24 + AS1411) + TMZ cargo and AsTNP (AS1411 + TGN) + PEG–PCL NP loaded with DTX. AsTNP (AS1411 + TGN) + PEG–PCL NP + DTX is a nanosystem comprising dual targeting aptamers, i.e., AS1411 aptamer for glioma targeting and the TGN phage-displayed peptide for BBB targeting, which are attached to poly(ethyleneglycol)-poly(ε-caprolactone) copolymers (PEG-PCL) NPs with DTX payload; it has demonstrated increased uptake into brain tumor and improved survival of GBM-bearing mice [4].

Nanospheres loaded with DOX and conjugated with AS1411 aptamer can selectively target tumor cells overexpressing NCL proteins and have proven increased efficiency in drug loading, delivery and persistence in tumors, as well as improved metabolic stability and biocompatibility [96,97]. The nanoconjugate delivery system AS1411 PGG-PTX designed from AS1411 aptamer and (*L*-*γ*-glutamyl-glutamine)-paclitaxel (PGG-PTX) has proven augmented uptake and retention within GBM cells, prolonged blood half-life, as well as increased median survival of the GBM-bearing animals, in comparison to the uncoated PGG-PTX [97,98]. In addition, synergistic aptamer–peptide drug conjugates, which combine in certain molar ratios the AS1411 with a peptide preloaded with chemotherapeutics (such as DOX and camptothecin CPT), have achieved higher potency at lower drug concentration than maximum tolerated dose [47].

Bi-functionalized conjugates were designed by coating micelles loaded with DTX and gadolinium (Gd) with AS1411 and an aptamer specific to transferrin receptor (TfR). The final conjugates showed a sustained release profile of DTX and Gd within 3 days, a significantly higher accumulation within mice brain and a good tumoral cytotoxic effect, as well as a lower *IC50* and longer *AUC*, than Taxotere^®^ [98,99].

Another brain tumor-targeting system transfected AS1411-decorated macrophage exosomes to silica NPs loaded with catalase and a sensitizer and it was tested in sonotherapy; it reduced tumoral hypoxia in vitro and in vivo due to exclusive intratumoural conversion of hydrogen peroxide to O_2_ [33].

Nanovesicles mimicking exosomes coated on surface with cholesterol-poly-conjugated AS1411 aptamer and loaded with PTX have proven efficient delivery of the chemotherapeutic drug into tumor cells through a murine dendritic cell membrane model [8,20,22].

Aptamer Gint4.T, targeting the human platelet-derived growth factor receptor PDGFRβ ectodomain overexpressed in GBM, inhibits in vitro GBM cell migration and proliferation and decreased in vivo tumor growth and differentiation [100]. Gint4.T conjugated to polymeric NPs containing a PI3K-mTOR inhibitor exhibited increased brain uptake in murine intracranial U87MG tumor xenografts than only drug-loaded NPs. Gint4.T conjugated on the surface of the nanocomplexes of poly(lactico-glycolic)-block-polyethylene glycol (PLGA-b-PEG) loaded with dactolisib (a dual PI3K-mTOR inhibitor) enabled quick, efficient (6500 times higher) in vitro accumulation inside PDGFRβ-overexpressing cells and 1000 times higher tumoral toxicity of drug, within 40 min of incubation, compared to its simple addition to cultured cells medium [88]. The principal results and limitations of Gint4.T Ap-nanoconjugates, such as Gint4.T–Nanopolymer (PI3K/mTOR inhibitor), TMSN@siHDGF–Gint4.T, Gint4.T/U2–NP conjugates, and Gint4.T–tFNA–DOX are also presented in Table 4.

TMSN@siHDGF-Gint4.T, a Gint4.T aptamer functionalized with chimera-capped silica NPs loaded with temozolomide (TMZ) and small interfering RNA hepatoma-derived growth factor (siHDGF), is a synergistic aptamer conjugate designed as a nanovehicle to co-deliver gene-drug siHDGF and TMZ in GBM therapy. In vivo, TMSN@siHDGF-Gint4.T significantly inhibited GBM growth and expanded survival time of GBM-bearing mice. In systemic administration, TMSN@siHDGF-Gint4.T protected siHDGF against enzymatic degradation due to its spherical nucleic acid-like spatial shape, ensured BBB permeability, and bound specifically to GBM cells with relevant intracellular uptake rate. After intratumoral internalization, TMSN@siHDGF-Gint4.T suffers sequential lysosomal cleavage and rapidly releases firstly siHDGF, which exerts its gene-silencing action, followed by slow release of encapsulated TMZ whose cytotoxicity is further enhanced by the previous HDGF downregulation [22,100]. In another study, Gint4.T conjugated to a STAT3 siRNA amplifies knockdown of STAT3 and its target genes, decreases GBM proliferation and neovascularization, compared to free STAT3 siRNA, as tested in cell culture and after in vivo administration to GBM mouse model [91,101,102].

The nanoconjugate obtained by coating TMZ- and *O*6-benzylguanine-loaded exosomes with peptide aptamer anti-angiopep-2 peptide and RNA aptamer anti-CD133 has significantly increased the survival rate in GBM-xenografted mice compared with free drugs [102]. Another Ap-NP example resides on the DNA aptamer conjugate with cisplatin AptBCis1 that crosses the BBB and binds to NCL, Na^+^-dependent excitatory amino acid transporters or solute carrier family 1 member 2 EAAT2/SLC1A2, and human Y-box binding protein 1 YB-1 proteins, which are overexpressed on lung cancer leptomeningeal carcinomatosis (LM). At doses of 1 mg/kg (corresponding to 0.35 mg/kg cisplatin) administered in LM mice models, this Ap-nanoconjugate has greater anti-tumoral effect than 2 mg cisplatin/kg and showed a 90% reduction of platinum levels in CSF [103,104]. Moreover, in breast cancer brain metastasis, a bifunctional aptamer with non-covalently intercalated DOX and targeting TfR and EpCAM passes through the BBB by transcytosis and is specifically and selectively internalized into tumor cells, where it delivers DOX in a pH-dependent manner; it also enables a greater tumoral uptake and retention of DOX than free drug administration [81,104].

Other preclinical cell-SELEX-selected DNA Ap-nanosystems for GBM discussed in Table 4 are RBT@MRN-SSTf/Apt mesoporous Ru-NPs, sgc8-DNA-dendrimer, and U2–AuNP. The DNA aptamer U2, similar to CL4 and CL32, targets EGFRvIII and inhibits tyrosine kinase autophosphorylation, thus inhibiting tumor growth, invasion and migration. Nanosystem U2–AuNP, designed from U2 and gold NPs as a radiation sensitizer, significantly accumulates within tumors and extends the survival period of GBM-bearing *Balb/c* nude mouse, in comparison to unconjugated gold NP [57]. Other examples are GBI-10 targeting malignancy-related tenascin-C in human GBM; and S6-1b that selectively targets human glioma cell line SHG44 and has shown uptake in GBM mice within 4 h after intravenous (i.v.) administration [76,91].

**Table 4:** Comparative insights into the main preclinical studies on Ap and Ap-NP conjugates tested in GBM models.

Ap-Nanoconjugate	Major Findings	Limitations	Ref.
AS1411–PGG–Paclitaxel	Enhanced PTX delivery, BBB penetration, solubility and pharmacodynamics; reduced systemic toxicity	Potential immunogenicity; need detailed PK/PD and data on efficacy variability due to NCL expression’s heterogeneity	[8,20,57]
AS1411–DOX Nanosphere	Improved tumor uptake and growth inhibition in U87 xenografts; low systemic toxicity	Lack of biodistribution and long-term toxicity analysis	[8,22]
AS1411–NP loaded with PTX	Preclinically advanced; improved BBB crossing and in vivo tumor growth control	Scarce data on the efficacy dependency on NCL expression’s heterogeneity	[8,22,76]
AsTNP (AS1411 + TGN) + PEG–PCL copolymers NP loaded with DTX	Dual-targeting BBB and tumor; improved brain delivery and uptake in glioma; reduced off-target toxicity; prolonged survival in mice	Complexity of dual modification; unclear stability in circulation; no human studies	[57]
tFNA (GS24 + AS1411) + TMZ cargo Tetrahedral DNA NP	Dual aptamers; enhanced BBB permeability and apoptosis in orthotopic glioma model; overcomes TMZ resistance;	Needs comprehensive PK/PD analysis; DNA structure stability in vivo; manufacturing complexity	[22]
AS1411–siRNA Nanoconjugates	Modular design and potential CNS-targeted delivery	Stability of siRNA and biodistribution challenges; limited in vivo data on BBB delivery efficiency and on off-target effects; scalability problematic	[8]
Gint4.T–Nanopolymer (PI3K/mTOR inhibitor)	High specificity to PDGFRβ; effective BBB crossing; in vivo tumor accumulation and potent tumor reduction	Requires further safety, immunogenicity and long-term efficacy studies; unclear reproducible synthesis and manufacturing scalability	[101]
TMSN@siHDGF–Gint4.T	Specificity and therapeutic versatility; potential *HDGF* silencing and tumor growth inhibition	Preclinical stage only; need endosomal escape optimization; limited data on: siRNA stability, potential off-target genes’ silencing and safety	[57,100]
Gint4.T/U2–NP conjugates	Preclinically advanced; enhanced targeting; high specificity; BBB penetration; in vivo efficacy; radiosensitization potential	Safety/toxicity unknown; scalability untested	[57]
Gint4.T–tFNA–DOX	Specific targeting of PDGFRβ+ glioma cells; effective in vitro and BBB penetration (U87MG GBM model)	Requires long-term efficacy studies and pharmacokinetic analysis	[101]
U2 Aptamer (EGFRvIII-targeted)	High binding affinity; increased radiosensitivity and reduced tumoral cell migration (U87-EGFRvIII GBM model)	Target specificity may limit applicability to EGFRvIII+ tumors only	[104]
U2–AuNP	Enhanced BBB penetration; inhibited DNA damage repair; improved survival in GBM-bearing mice; selective radiosensitization	Limited systemic toxicity and PK data; Long-term biodistribution unknown; Optimization needed for targeting efficiency	[57,104]
RBT@MRN-SSTf/Apt Mesoporous Ru-NPs	Dual targeting: *AS1411* + *TfR* Ap + photosensitizer; deep tumor penetration and extended survival in vivo (U87MG mouse model)	Limited to laser-accessible tumors; Photodynamic therapy (PDT) safety and dosing need assessment; Ru-NPs immunogenicity risk	[33,72]
sgc8-DNA-dendrimer	High specificity (xenograft models); High-affinity conjugation potential; Well-characterized conjugation chemistry; Self-assembled modular nanostructures	Lacks in vivo validation; target expression (PTK7) is not glioma-specific, limiting translational relevance; unknown BBB permeability	[20]
D-siGFP (PAMAM–siRNA conjugate)	Proof of concept for GBM siRNA delivery (orthotopic GBM in CX3CR-1GFP mice); enhanced siRNA stability and T1/2; 30% in vivo tumor macrophage uptake	Limited data on PK, in vivo stability and long term toxicity	[105]
CTLA4Apt–STAT3 siRNA	Dual targeting of immune checkpoint and *STAT3* oncogene suppression; specific immune modulation	Complex delivery system; scarce glioma-specific efficacy, immunotoxicity and PK data	[59,68]
A32–Quantum Dot (QD-Apt)	Tumor visualization in glioma-bearing mice (orthotopic U87-EGFRvIII model); fluorescence-guided surgery	Potential QD toxicity; challenges in clinical imaging translation	[106]

### 5.2. Aptamer–siRNA Chimeras

The delivery of siRNA by aptamers is based on the formation of stable duplexes by hybridization between the complementary regions [95]. The aptamer–siRNA chimera D-siGFP is obtained by covalent conjugation of hydroxyl-terminated poly(amidoamine) (PAMAM) dendrimer to siRNA against green fluorescent protein (GFP) using a glutathione-sensitive linker. When administered intratumorally to orthotopic multiform GBM in CX3CR-1GFP mice, D-siGFP have proven significant GFP knockdown in vivo (∼30%), increased enzymatic stability and T1/2 of cargo siRNA, preserved ability of PAMAM dendrimers to passively target reactive microglia, enhanced distribution into tumor parenchyma and tumor-associated macrophages, and minimized off-target effects in other cell populations [105]. The main strengths and limitations of D-siGFP are discussed in Table 4.

Other aptamer–siRNA chimeras could be illustrated by the following: (a) AS1411–siRNA Nanoconjugates (discussed in Table 4); (b) the bi-specific aptamer based on AS1411 targeting NCL and a “CpG” oligodeoxynucleotide targeting Toll-like receptor TLR9 that was designed to deliver siRNA specific to osteopontin mRNA, which is highly expressed in GBM [10,107]; (c) AS1411 aptamer conjugated with SMG1 RNAi, which has proved to be an efficient and well-tolerated enhancer of immune response against various tumors, including CNS ones [108]. In addition, anti-GD2 aptamer selectively delivers into GD2+ neuroblastoma tumors the conjugates of the neuroblastoma-specific *MYCN* siRNA and DOX; the *MYCN* gene encodes the human proto-oncogene protein N-Myc (N-myc or basic helix-loop-helix protein 37 bHLHe37) [73]. The effects exhibited by this aptamer-siRNA conjugate were *MYCN* gene silencing, tumor growth reduction and lack of toxicity even at chemotherapy doses doubled over standard ones [10]. Moreover, aptamer-mediated delivery of siRNA specific against the junction site of two mRNA within the fusion oncogenes, which are prevalent and associated to poor prognosis in GBM and other neurologic tumors, has proven, in vitro, no effects on physiological protein expression [47,109]. Multivalent aptamer-siRNA conjugates obtained from aptamers specific to mucin-1 and siRNA against *bcl-2* genes, also loaded with DOX by intercalating it within nucleic acid sequences, were designed to target drug-resistant cancer cells [29]

### 5.3. Aptamer-Antibody Conjugates (Immunotherapeutical Ap, “Oligobody”)

Aptamer-antibody conjugates or “oligobody”, such as aptamer against VEGF linked to anti-cotinine antibody, anti-EGFR aptamer linked either to anti-epidermal growth factor receptor 2 (ErbB2) compact antibody or to an immunomodulatory (anti-PD-L1) antibody, have promising distribution into solid tumors, activate T cells against tumor cells and increase anticancer efficiency [10,22]. Immunotherapeutic aptamers aim to (1) inhibit the action of the immunosuppressive cytokines (like transforming growth factor TGF beta, interleukin IL10) produced within TME by tumor-infiltrating lymphocytes; or (2) to antagonize the main immune checkpoint receptors (like T-cell immunoglobulin and mucin domain 3 TIM-3, programmed cell death 1 PD-1, programmed cell death ligand 1 PD-L1, cytotoxic T-lymphocyte-associated protein 4 CTLA-4); or (3) to co-stimulate T lymphocytes or antigen-presenting cells by binding to receptors such as CD40, CD28, OX40, 4-1BB [11,110].

For instance, the RNA aptamer R5A1 is targeted to IL-10 receptors; the agonistic RNA aptamers CD28Apt2, CD28Apt7 and 4-1BB conjugated to 2′-fluoropyrimidine stimulate cellular immune response, extend survival of tumor-induced mice, and 4-1BB additionally increases the therapeutic index of immunotherapy with osteopontin or VEGF mAbs [90,108]. Among antagonistic aptamers, CTLA4apt–STAT3 siRNA, specific to CTLA4 and conjugated to *STAT-3* siRNA, inhibits tumor proliferation and metastasis by down-regulation of *STAT-3* gene expression in CD8+ and regulatory T cells infiltrated within tumors; its main strengths and limitations are presented in Table 4 [59,69]. The DNA aptamer specific to PD-L1, aptPD-L1, stimulated IFN-γ, IL-2, C-X-C motif chemokines, TNF-α, proliferation of cytotoxic and helper T cells infiltrated in tumours and also inhibited in vivo tumour growth [69,101]. The anti-proliferative aptamers bi-(AID-1-T) and bi-(AID-1-C) have proven synergistic and persistent down-regulatory effects on the migratory potential and on the expression of *c-Myc*, *PARP1*, nestin, *L1CAM* and caveolin-1 genes, in human GBM cell cultures [111]. Either as monotherapy or combined to radiotherapy, the TIM-3 aptamer induced a strong specific immune reaction by increasing the ratio of proinflammatory T lymphocytes CD8+ to T regulatory lymphocytes (Treg) in the TME and myeloid cells, leading to greater overall survival in mouse models of pediatric diffuse midline gliomas, in comparison to control groups [112].

The main results and limitations of other preclinical studies which have been performed on aptamer-antibody conjugates in gliomas, GBM, and neurodegenerative diseases could be summarized as follows:

➢ EGFR GL21.T Ap–Cetuximab conjugate—explored in GBM, offers the advantages of enhanced EGFR targeting, dual specificity and improved GBM uptake; however, it implies a complex synthesis and immunogenicity risk (due to Ab fragment) [61,113,114];
➢ AS1411–Antibody Dual Targeting System—investigated in gliomas, combines Ap specificity with Ab-dependent cell-mediated cytotoxicity and has proven enhanced BBB permeability; the limitations are related to its stability, scalability and the optimization for in vivo delivery [61,80,115];
➢ Aptamer–Trastuzumab Fusion (HER2+ brain tumors)—tested in GBM/brain metastases, has demonstrated improved tumor uptake, but its efficacy is limited by HER2 expression heterogeneity; in addition, it has potential immunogenicity [6,30,113];
➢ RNA Aptamer–Anti-Aβ Antibody Hybrid—investigated in AD, has inhibited fibrillar aggregation and immune clearance and has shown strong amyloid plaque reduction in vivo; however, its long-term efficacy is unclear, and it might induce undesired inflammatory reactions [80,115,116];
➢ Anti-Tau Antibody–Aptamer Bioconjugate—investigated in AD, has proven the advantages of strong targeting and inhibition of Tau aggregation, as well as improved brain delivery; its current limitations reside on the in vivo degradation risk [80,114,116].

### 5.4. Ap-NP as Radiotherapy Enhancers

Aptamers as radiotherapy enhancers could ameliorate the high radio-resistance of GBM and the impermeability of the BBB against radiosensitizers. They could be exemplified by aptamer TDSGNPs, based on PEG-functionalized silver-gold core-shell NPs targeted both to TfRA4 on the BBB and DNA1 on GBM cells; TDSGNPs demonstrated in vivo delivery across the BBB, an GBM uptake peak after 3 h i.v. injection, extended median survival of mice, and superior radiosensitizer effect than NPs alone [117]. Moreover, the nanoconjugate comprising the aptamer GMT8 on the surface of PEG-coated Ag@Au core-shell NPs has increased by nine times the uptake inside U87 tumor cells than NP without aptamer and has also expanded mean survival time (to 58.5 days from 36.5 days) of GBM xenografted mice than radiotherapy alone [118].

### 5.5. Comparative Insights into the Main Preclinical Studies on Ap and Ap-NP Conjugates Tested in GBM Models

**Figure 3:**
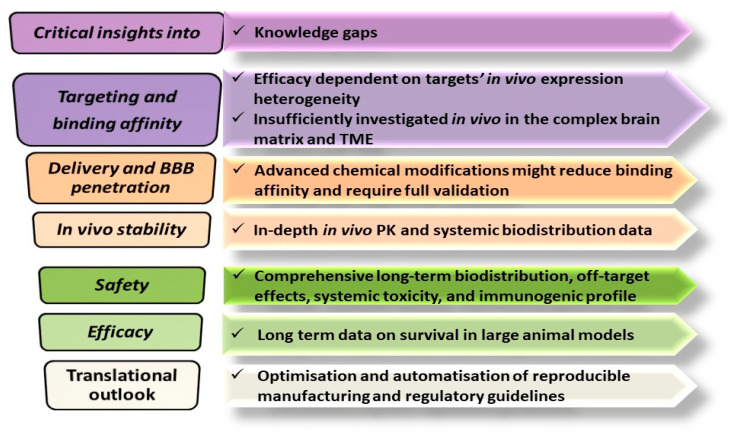
Critical insights into Ap-functionalized NP preclinically tested in GBM models.

Active Ap-nanoconjugates based on AS1411, Gint4.T and U2 show high tumor specificity, effective BBB penetration and tumor growth suppression (Table 4). RBT@MRN-SSTf/Apt Mesoporous Ru-NP is promising in combined chemo–PDT (photodynamic) therapy. tFNA–TMZ conjugate showed efficacy in TMZ-resistant models, highlighting potential to address glioma drug resistance. Dual-targeting strategies (e.g., BBB and tumor cell biomarkers) offer enhanced delivery, but entail higher structural complexity. Critical insights into Ap-functionalized NP preclinically tested until now for neuro-oncological purposes are summarized in Figure 3 [22,33,57,72,76,104,109].

## 6. Preclinical Aptamer-Nanoconjugates in Amyloidopathies

### 6.1. Description and Comparative Analysis

Specific antiamyloid aptamers have proven to be a cost-effective and non-immunogenic alternative to immunotherapeutic mAb, due to their small size, facile and large-scale chemical synthesis, high stability, and low immunogenicity [14,31,58,119]. There are some barriers in the aptamers’ development for amyloidopathies which are related to difficulties in the aptamers’ selection due to the heterogeneity and conformational metastability of the oligomers of amyloidogenic proteins, as well as to Ap’s targeted delivery to amyloidogenic proteins into brain parenchyma. These barriers could be avoided by stabilization of targeted aptagens (by covalently tethering of the protein monomers to more stable forms mimicking the metastable oligomers), and the application of both advanced high-throughput next-generation sequencing and structural prediction software, which will be coupled with artificial intelligence and machine learning algorithms, in order to increase the yields of selected aptamers with high binding affinity and specificity [20,119].

RNA aptamers E22P-AbD4, -AbD31, and -AbD43 preferentially bind to Aβ42 protofibrils due to the formation of a G-quadruplex structure and inhibit in a dose-dependently manner their neurotoxicity in SH-SY5Y neuroblastoma cell line. Other experiments on F5R1 and F5R2, 58-nucleotide DNA aptamers conjugated to a peptide carrier CADY (CADY is a cell-penetrating peptide with the sequence GLWRALWRLLRSLWRLLWRA), have proved that they have high binding affinity and they inhibit both αSyn aggregation and the synaptic and neuronal destruction caused by αSyn overexpression [63].

The main results and limitations of some preclinical studies on Ap and Ap-NP conjugates tested in amyloidopathies are discussed in Table 5 for the following: E22P-MAbD4, -MAbD31, -MAbD43; BI1/B1-CT; F5R1 exosomes; Au@PDA–Apt NPs; Aβ-Apt; as well as for aptasensors Aβ_40_ Aptamer–Silicon FET Sensor and Micromotor AuNP-Aptamer Assay [20,26,29,51,56,62,63,104,116,120].

**Table 5:** Comparative insights into some preclinical studies on Ap and Ap-NP conjugates tested in amyloidopathies.

Ap-Nanoconjugate	Major Findings	Limitations	Ref.
RNA aptamers E22P-MAbD4, -MAbD31, -MAbD43	Binds Aβ_42_ protofibrils; detects oligomeric aggregates in AD mouse brains (Tg2576/PS2)	No demonstrated therapeutic benefit; unknown clearance	[20]
BI1/B1-CT (BACE1-targeting)	Reduces Aβ_42_ levels; improves cognition in Tg6799 mice AD model via i.c.v. (intracerebroventricular) injection	Invasive delivery; scarce data on long-term effects	[104]
F5R1 Ap for α Synuclein in RVG tagged exosomes	Specificity for fibrillar α Syn over monomers; BBB crossing and CNS-targeted delivery; mitigated αSyn aggregation; improved motor functions outcomes in a robust synucleinopathy mouse model	Exosomes’ scaling-up production challenging (genetic engineering of the exosomes); limited data on Ap stability, release kinetics and off-target payload delivery	[63]
Au@PDA–Apt NPs	Dual-action: inhibits Aβ_1−40_ aggregation and disaggregates mature Aβ_1−40_ fibrils in vitro; protection against Aβ-induced neuronal membrane damage; reduced cytotoxicity; multifaceted biophysical validation methods	unknown toxicity/immunogenicity; unclear BBB crossing to reach amyloid deposits in brain tissue	[26,51]
Aβ-Apt (DNA aptamer)	Completely inhibits Aβ_42_ fibrillation in in vitro aggregation assays; selective oligomer binding	No in vivo data; potential degradation and poor bioavailability	[29]
Polypeptide Based Multimodal Nanoconjugates (polyglutamate + neuroprotective agents + Angiopep 2)	In vivo efficacy in a transgenic AD APP/PS1 mice model, including functional behavioral outcomes; Increased dendritic density, reduced β amyloid aggregates; rescued memory and olfactory deficits	No Ap-based system (peptide small-molecule targeting); unaddressed long term safety, bioaccumulation and immunogenicity	[62]
Aβ_40_ Aptamer–Silicon FET Sensor	In vitro sensor platform; high sensitivity (detects Aβ_40_ at 0.1 pg/mL without labeling)	Diagnostic only; lacks clinical validation for real-world use	[120]
Micromotor AuNP-Aptamer Assay	Rapid (5 min) and sensitive detection (0.10 pg/mL) of Aβ_42_ in CSF/plasma of AD patients	complex integration into diagnostics	[116]

Anti-Aβ42 DNA aptamer Aβ7-92-1H1 binds selectively and with higher affinity to Aβ42 oligomers than to Aβ42 monomers, also to Aβ40 or other amyloids, and inhibits Aβ42 fibril formation [121]. Aptamer a-syn-1 exhibited affinity for monomeric αSyn and a strong inhibitory effect on αSyn aggregation and dissemination, which is a neuropathological hallmark of PD. In transgenic mice overexpressing the human *A53T* variant of *αSyn*, a-syn-1-loaded liposomes were delivered into the brain by repeated daily i.p. injections; they reduced aggregated αSyn levels in the prefrontal cortex, caudate and substantia nigra [122,123]. The G-quadruplex structured T-SO530 aptamer is selective against αSyn oligomers and its binding affinity is further increased by the addition of synthetic stabilizers of G-quadruplex (i.e., L1H1-7OTD, TmPyP4). The RNA aptamer DP7 was targeted to the human PrP90–129 domain critical for the conversion of PrPC into PrPSc and reduced PrPSc/PrPC ratio, as it was demonstrated in prion-infected neuroblastoma N2a murine cells [28,56].

The NXP031, a conjugate of vitamin C (VitC) with an aptamer able to bind and stabilize it, stimulated Aβ-degrading endopeptidase expression and proved a greater inhibition on Aβ accumulation than VitC; reduction of lipid peroxidation levels; upregulation of Nrf2-mediated antioxidant pathways; and downregulation of neuroinflammatory process. In mice intrahippocampally injected with *Aβ*, the dose-related neuroprotective effects of NXP031 consist of reduced *NOX-2* expression in the hippocampus (*NOX-2* gene encodes Nox2 protein, i.e., NADPH oxidase 2, also known as cytochrome b(558) subunit beta or cytochrome b-245 heavy chain); diminished neuronal apoptosis and synaptic degeneration; and attenuated memory decline [116,124].

In tauopathy-associated neurodegenerative diseases, the DNA aptamer BW1c, administered by retro-orbital injection, has demonstrated the ability to cross the BBB and to bind with high affinity (*Kd* = 6.6 nM) to monomeric Tau protein. In this way, BW1c efficiently inhibited Tau oligomerization and aggregation by arachidonic acid, as well as inhibiting Tau hyperphosphorylation GSK3β- and okadaic acid-mediated [8,22]. Another aptamer 3146 binds to Tau isoforms in the order 2N4R (*Kd* = 13 nM) > 0N4R (49 nM) > 0N3R (84 nM) > 1N3R (116 nM). RNA aptamer tau-1 binds to 2N4R-Tau, preventing the formation of 2N4R-Tau dimers and trimers, and was isolated by SELEX from a collection of 90-nucleotide RNA library with 40-nucleotide random region. The inhibition of Tau protein phosphorylation in the brain was also obtained by a bifunctional circular aptamer designed from transferrin aptamer able to cross the BBB by transcytosis and T4 enzymatic ligase aptamer, having increased half-life, brain delivery and good effect on the memory in mice models [29]. AptaGron is another aptameric system aimed to catalytically downregulate targeted proteins, such as Tau, NCL and eukaryotic initiation factor 4E (eIF4E), which cannot be achieved by specific small-molecule ligands. AptaGron is composed from peptide nucleic acid sequences with a *N*-degron peptide and a complementary base pairs segment and does not require synthetic conjugations between proteolytically units and aptamers [70].

### 6.2. Overall Critical Insights into the Main Preclinical Studies on Ap and Ap-NP Conjugates Tested in Amyloidopathies

The above-mentioned preclinical studies revealed the importance of Ap’s selectivity for Aβ oligomers (e.g., Aβ-Apt DNA aptamer) [29]. Although many of the above-mentioned aptamer systems have shown in vitro efficacy by strong inhibition of fibril formation, they require further optimization of the delivery vehicles. Moreover, only the RNA aptamers E22P-MAbD4, -MAbD31, -MAbD43 and BI1/B1-CT (BACE1-targeting Ap) have been tested in animal models, and even then, they are used primarily for detection or biomarker reduction and not for neurofunctional recovery [20,104,122,123]. Preclinical testing performed on the anti-amyloid aptamer nanoconjugates has shown promising in vitro efficacy and groundbreaking in vivo aptamer delivery (e.g., exosome mediated αSyn targeting). From a translational perspective, F5R1 aptamer for α Syn in RVG-tagged exosomes offers proof-of-concept for aptamer therapy in CNS conditions, reiterates the importance of specificity for fibrillar α Syn over monomers to mitigate αSyn aggregation, and is also promising for motor functional recovery; however, its next developmental steps should include PK and safety profiling and also scalable delivery systems. Au@PDA Apt NPs for Aβ offers strong in vitro foundation, but requires robust demonstration of brain delivery, tolerability and efficacy in animal models. Although not an Ap-derived system, Polypeptide Based Multimodal Nanoconjugates points to feasibility of modular nanoconjugates regarding BBB-targeting; however, substitution with aptamers might introduce new variables in stability and biodistribution [26,51,62,63]. In neurodegenerative contexts, aptamer–siRNA nanoconjugates are still in early proof-of-concept stages with no direct disease-specific validation yet [8].

All Ap-NP systems lack translation to clinical trials, due to current limited long-term safety, immunogenicity and dosing data. Moreover, the integration of molecular biomarkers and neurofunctional outcomes in prospective long-term, larger and randomized clinical trials would greatly support the translational success of Ap-NP conjugates in this therapeutic area [20,62,104]. Although gold nanoparticle–aptamer assemblies have been widely used in oncology for drug delivery and photothermal therapy, few platforms have crossed over into anti-amyloid therapy, highlighting limited cross-application and rigorous in vivo validation specifically for amyloid targets [62,63].

**Figure 4:**
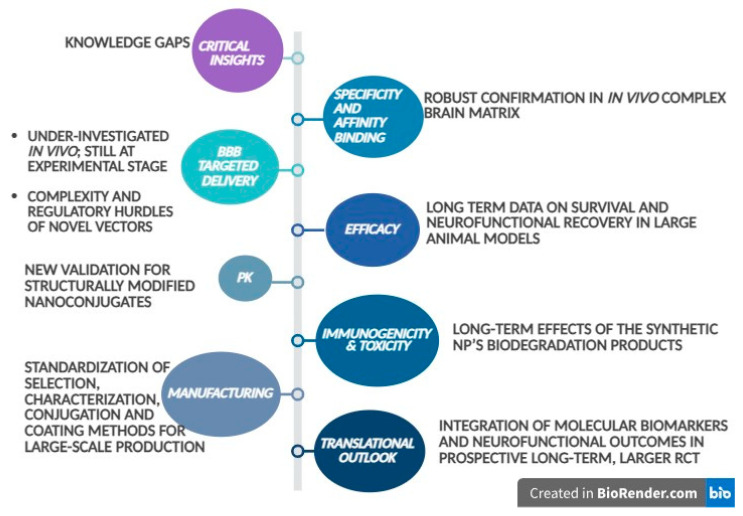
Overall critical insights into Ap-functionalized NP preclinically tested in amyloidopathies.

Major knowledge gaps remain in BBB penetration, safety, mechanistic clarity, and scale-up manufacturability. For instance, most effective systems for BBB-targeted delivery are still in the experimental rather than the translational stage. Although transferrin-targeting strategies are promising, studies demonstrating effective delivery through the BBB of the Ap-functionalized NP are still under-investigated in vivo. In addition, novel delivery vectors bring complexity and regulatory hurdles and are far from clinical maturity. Further validation of structurally modified nanoconjugates should assess their long-term effects in the in vivo complex brain matrix, effects, and the risk of immune activation, especially by the synthetic NP’s biodegradation products, as well as the deeper intracellular mechanisms of the anti-amyloid effect. Concerning manufacturing and scalability, the following aspects have to be established: standardization of selection and characterization methods, including negative controls, replicates, affinity metrics; standardization of covalent aptamer conjugation methods to improve batch-to-batch consistency; the alignment of the coating of exosomes with RVG peptide aptamer to current large-scale production standards [20,62,64,104,122,123]. Overcoming these challenges is essential for clinical translational impact of the antiamyloid Ap-nanoconjugates. Critical insights into Ap-functionalized NPs tested until now in amyloidopathies are briefly illustrated in Figure 4.

## 7. Preclincal Aptamer-Nanoconjugates in Multiple Sclerosis (MS)

### 7.1. Description and Comparative Analysis

The most relevant Ap-functionalized NPs preclinically tested until now in MS models are comparatively analyzed in Table 6: ApTOLL, Myaptavin 3064, and Exo APT (LJM-3064 Exosomes). ApTOLL is an aptamer selected to antagonize Toll-like receptor 4 (TLR4), which is involved in immunological and inflammatory reactions in demyelinating diseases like multiple sclerosis (MS) and autoimmune encephalomyelitis (EAE). ApTOLL demonstrated anti-inflammatory, neuroprotective and remyelinating effects on an ex vivo model of cultures of murine and human oligodendrocytes demyelinated with lysolecithin, and also a positive influence on the clinical signs of EAE in mice [125,126]. Moreover, in mice with permanent ischemic stroke and in early phase clinical studies, ApTOLL proved superior cerebroprotective activity and better tolerability than TAK-242 (a TLR4 inhibitor), due to its modulatory effects on circulating leukocytes and on local immune reactivity; its accumulation into brain ischemic regions; its reduction effects on the infiltrating neutrophils density; and on edema volume, thus ameliorating neurofunctional disturbances and hemorrhagic risk [126].

Myaptavin-3064, a biotinylated DNA aptamer conjugated to streptavidin, rich in guanosine and adopting a G-quadruplex structure, specifically targets myelin from oligodendrocytes with binding affinity similar to mAb IgM and stimulates remyelination in a murine model of MS [127]. The aptamer LJM-3064, from which the previous Myaptavin was designed, is attached to the surface of exosomes derived from murine mesenchymal stem cells creating the conjugate Exo-APT. Exo-APT is used both as a targeting vector to oligodendrocytes and as a therapeutic agent due to its remyelinating action. Exo-APT strongly suppresses demyelination, neuroinflammation and lesion severity, as demonstrated after i.v. administration in mice models of MS or EAE, and also in vitro on oligodendrocytes cell lines (OLN93) [34].

Another Ap-nanosystem uses C4-3 aptamer to target NPs to neurons, because C4-3 acts as an agonist to tropomyosin receptor kinase B (TrkB), which is overexpressed by neurons, being activated by BDNF (brain-derived neurotrophic factor). In primary cultures of embryonic rat cortical neurons, C4-3 aptamer stimulated the active/phosphorylated form of the receptor and demonstrated a key role in neuronal survival, regeneration and plasticity [64,128].

**Table 6:** Comparative insights into the main Ap-Np conjugates preclinically tested in MS.

Ap-Nano Conjugates	Myaptavin-3064	ApTOLL	Exo-APT *(LJM-3064 Exosomes)*	Ref.
Main results	BBB-permeable and increased brain uptake; selective to differentiated human oligodendroglioma cells; stimulates remyelination in chronic demyelination model; functional secondary structure in physiological conditions	Reduces inflammation and demyelination mouse models; improves remyelination; enhances motor function, oligodendrocyte generation; favorable PK, safety profiles in previous stroke and healthy volunteer trials	Promotes in vitro proliferation of OLN93 oligodendroglial cells; reduces in vivo inflammation and demyelination; improved clinical EAE scores in mice	[32,33,34,52,125,126,127]
Strengths	In vivo remyelination efficacy: robust histological evidence of repair in a well-established MS model. Mechanistic insight: oligodendrocyte targeting. PK confirmation: CNS distribution in inflamed tissue—a key translational milestone.	In vivo functional outcomes supported by behavioral and histological metrics. Dual mechanism: immunomodulation (via TLR4 antagonism in oligodendrocyte progenitor cell OPC) + remyelination; partial insight of downstream signaling mechanism; systemic Ap with known PK; strong translational outlook (advanced safety data in humans and efficacy in multiple MS models).	Clinical score improvements and lesion reduction in EAE based on functional in vivo results. Combines targeting and delivery platform (targeting specificity + biologically active carrier)	
Limitations	Partial mechanism (undefined downstream signaling); Translation hurdles: streptavidin-based multimerization-potential immunogenicity, regulatory complexity. Limited dosing/safety data: only single-dose studies, no longitudinal safety or off-target effects; In vivo structural variability (metastability = K⁺-mediated switching of LJM-3064 G-quadruplex)	Unknown downstream pathways promoting remyelination; Unclear dosing regimen (effective dose ranges and therapy duration in models); Comparative efficacy with Myaptavin-3064—unknown	Reproducible production and large-scale manufacturability of exosome and surface conjugation; unexplored long-term immune response profile; unknown detailed remyelination mechanism	
Stage of development	Preclinical with PK data	Preclinical + human safety data	Preclinical only	
Translational hurdles	Immunogenicity; multimeric scaffold	Detailed mechanism; dosing strategies	Production scaling; safety profiling	

### 7.2. Critical Insights on Preclinical Studies on Ap and Ap-NP Conjugates Tested in MS

Each of the main Ap-functionalized NPs preclinically tested until now in MS models offers unique advantages, with ApTOLL leading in translational potential, Myaptavin 3064 showcasing mechanistic promise, and Exo-APT pioneering targeted delivery. However, they all require deeper mechanistic clarity, safety evaluation, and manufacturing optimization to make meaningful progress toward human use.

A comparative analysis of the Myaptavin 3064, ApTOLL and Exo APT leads to the following conclusions: (1) mechanistic understanding varies widely—Myaptavin 3064 shows robust targeting, ApTOLL demonstrates dual effects, while Exo-APT needs further mechanistic studies; (2) delivery advancements show promise—ApTOLL and Exo-APT overcome BBB challenges more elegantly than streptavidin-based Myaptavin 3064; (3) translational readiness differs—ApTOLL is furthest along with human safety data, while Myaptavin 3064 and Exo-APT remain early-stage with larger safety/developmental gaps; (4) safety and standardization are missing—all Ap-NPs lack comprehensive long-term toxicity, immunogenicity and pharmacodynamics studies; (5) comparative efficacy is absent—direct comparisons in the same model across these Ap-NP systems would be highly valuable. Data supporting these conclusions are also presented in Table 6 [33,34,52,125,126,127].

## 8. Aptasensors Preclinically Tested in Brain Diseases

### 8.1. Aptasensors Preclinically Tested in Gliomas

Bio-imaging aims to visualize cell surface biomarkers, as well as in vivo intracellular processes, structures or molecules. Brain imaging aptamers conjugated to fluorophores and radionuclides can be exemplified by AS1411 targeted to glioma peptide TGN (TGN is a peptide of 12 amino acids TGNYKALHPHNG) and conjugated with fluorophore Cy3, as well as by AS1411 conjugated to cobalt-ferrite nanoparticles labelled with fluorescent rhodamine, which were tested as aptasensors in mice gliomas [106,117]. As a theranostic tool in brain glioma, the DiR-AsTNP targeted delivery system is constructed by conjugation of the following components: the AS1411 DNA aptamer, the dye indotricarbocyanine (DiR), the TGN peptide and PEG-poly(ε-caprolactone) (PEG-PCL) NPs. The role of TGN is to enhance the permeability through the BBB and the role of AS1411 is to deliver the entire Ap-NP conjugate inside the tumor with high uptake rates. Intracellularly, DTX encapsulated within aptamer-TGN particles will act as an inhibitor of microtubule depolymerization, while the encapsulated fluorescent dye (DiR) enables the optical in vivo bioimaging [129].

The aptafluorescence of the aptamer H02 targeting integrin α5β1, that is involved in GBM angiogenesis and aggressiveness, has enabled the differentiation of the expression levels of α5 subunit among 10 human GBM cellular types and has been 78% correlated to antibody immunofluorescence [130].

Moreover, ATP-responsive aptamers were conjugated to brain cell-derived exosomes and they were delivered across the BBB through receptor-mediated transcytosis, acting as aptasensors and in vivo noninvasive imaging agents for various brain metabolites. These aptasensors proved high permeability through the BBB and high brain uptake efficiency, and also provided spatial distribution and dynamics of the ATP levels (which unevenly decrease throughout the brain during neurodegeneration) within living, intact, heterogenous brain cells and parenchymal architecture. Further optimization will address their specificity to ATP, as they are currently binding to other adenosine compounds (ADP, AMP) which are present in lower concentrations than ATP [131].

The aptamer TTA1 specific to the extracellular matrix protein tenascin-C and labeled with fluorescent rhodamine RedX or radionuclid ^99m^Tc, has demonstrated, after i.v. administration in GBM-xenografted models, rapid perivascular delivery (10 min), quick tumoral uptake (maximum 1 h), good diffusion into tumoral parenchyma (within 3 h), long persistence in tumor (18 h), a high tumor-to-blood ratio (50) within 3 h, and rapid clearance from systemic circulation [10,132]. The QD-aptamer QD-Apt is a complex comprised of aptamer A32 targeting EGFRvIII overexpressed in many human gliomas and of the fluorescent quantum dots. QD-Apt has demonstrated low adsorption on plasma proteins, reduced toxicity in vitro and in vivo, high permeability through the BBB, and selective uptake in gliomas with an intense fluorescent signal, thus enabling intraoperative clear visualization of macroscopic tumor borders (Table 4); therefore, QD-Apt provides excellent surgical guidance as bioimaging agent (Table 4) [71,106].

Aptamer AS1411 conjugated to a DNA octahedron acts for simultaneous fluorescent bioimaging of 2 tumor mRNAs, proving selective and increased uptake into cancer cells. Another ^99m^Tc-labeled aptamer with specificity for human matrix metalloprotease 9 was used for real-time tumor slice monitoring. Another nanoconjugate designed from the DNA TLS11a aptamer conjugated to gold and platinum NPs has been used as a cytosensor for tumoral cells [133]. An original method called ‘DNA origami traffic light’ aims to incorporate an ATP-sensitive aptamer coupled with dyes on two sides of the structure of DNA origami; this product will detect energy transfer emissions after the interaction with ATP [134].

In addition, aptamer apHAT610 is a promising tool, superior to monoclonal antibody diagnostics, in aptahistochemistry of meningiomas overexpressing histone acetyl transferase-1 (HAT1), which are among the most aggressive meningiomas, with poor prognosis and higher rates of early relapse [135].

In brain cancers, other safe and effective theranostic agents can be illustrated by chitosan-graft-*d-α*-tocopherol PEG 1000 succinate (TPGS-chitosan) copolymers and chitosan-PLGA NPs, which encapsulate DTX and upconversion nanoparticles (UCNP), these co-delivery tools being functionalized on the surface with both AS1411 aptamer and arginine-glycine-aspartate (RGD) [98,136]. RDG is the smallest and most highly preserved fibronectin sequence able to bind to the αvβ3 integrin receptor overexpressed on the TME. These targeted co-delivery theranostics have shown high encapsulation rates and sustained release of DTX, increased uptake in tumor cells and cytotoxicity at lower DTX concentrations, significant tumor growth suppression in murine models, good tolerability, enhanced bioavailability, and high persistence of DTX in brain tumors and blood [99,136]. Furthermore, multiple aptamers AS1411, RGD and TTA1 were conjugated to MNP@ SiO2(RITC)-PEG/COOH/pro-N/NH2 NPs, as aptasensors for various primary brain tumor cell lines and brain metastases [114].

### 8.2. Aptasensors Preclinically Tested in Neurodegenerative Diseases

aptaEGFET is an electrochemical aptasensor dedicated to detecting BDNF and to early-diagnose and clinically monitor neurodegenerative diseases. aptaEGFET combines the binding specificity of the DNA aptamer with powerful electrical features of reduced-graphene oxide field-effect transistors (r-GO-FETs) and with electrodes of gold and silver chloride. aptaEGFET might be a cost-effective and reliable alternative to traditional methods (ELISA: enzyme-linked immunosorbent assay and electrochemiluminescence), due to its high selectivity, specificity, sensitivity (limit of detection of 0.4 nM BDNF), broad linear response domain (from 0.025 to 1000 nM) and stability in the complex brain matrix [137]. In addition, NV_B12 aptamer has significant binding affinity in a dose-related manner and selectivity for detecting BDNF and might also become an alternative to mAb [93].

Innovative aptamer-based surface-enhanced Raman scattering (SERS) biosensors integrated with a multiwell glass chip enabled a selective, sensitive (atto-molar blood levels), dynamic and quantitative correlation with some biomarkers for early-stage AD diagnosis and progression, such as neurogranin (Nrgn), angiopoietin-2 (Angio-2), PRDX3, lactate dehydrogenase (L-LDH), and Tau τ-441 [138]. Capillary electrophoresis-based systematic evolution of ligands by exponential enrichment (CE-SELEX) was used for the selection of these high-affinity aptasensors [63,138,139]. Other aptasensors conjugate highly specific aptamers for Aβ40 and Aβ42 biomarkers to a simple, rapid and affordable CRISPR-Cas12a-based fluorescence sensor and they might be an accurate, sensitive (1 pg/mL Aβ40 and 0.1 pg/mL Aβ42), quick (1 h) and early AD diagnostic tool, superior to traditional ELISA [120]. DNA aptasensors for amyloid beta Aβ, Tau (τ), and αSyn, coupled with an at-home, wireless graphene field-effect transistor biosensor platform, have proven great precision and sensitivity (detection limits of 10 fM, 1-10 pM, 10-100 fM for Aβ, τ, and αSyn, respectively), the absence of cross-reactivity, as well as the ability to discriminate between neurodegenerative (AD, PD) and normal brains [140]. Moreover, gadolinium (Gd)-dodecane tetraacetic acid DOTA-ob5 aptamer as a contrast agent specific to the AD biomarker oligomeric amyloid beta (oAβ) has shown maximal signal in 20 min after its injection in the bilateral hippocampus, thalamus and amygdala, in different AD models, thus becoming a promising aptasensor in AD’s early diagnosis and progression monitoring [141].

## 9. Ap-NP Conjugate Clinical Translation

There is no currently approved Ap-NP therapy for gliomas or neurodegenerative disorders. Ongoing clinical investigations involving Ap-based therapeutics tested in these disorders are presented in Table 7: GLORIA for Olaptesed pegol in GBM; ApTOLL for anti-TLR4 DNA aptamer in stroke and MS; and NU 0129 for NA/Au NP targeting siRNA against *BCL2L12* gene in recurrent GBM.

Olaptesed pegol (NOX-A1), a pegylated Spiegelmer and chemokine CXCL12-neutralizing aptamer, inhibits CXCL12-CXCR4 axis (CXCL12, stromal cell-derived factor-1; CXCR4, C-X-C Motif Chemokine Receptor 4) responsible for tumor growth, angiogenesis, immune suppression and intercellular crosstalk within TME, in refractory GBM and recurrent GBM post-radiotherapy. The *L*-RNA aptamer’s Spiegelmer design resists nucleases and has minimal immunogenicity; signals showed reduced tumor vascular perfusion and meaningful plasma levels [142].

In a dose-escalation GLORIA phase I/II trial for GBM (grade 4 WHO) incompletely resected patients, olaptesed pegol, administered i.v. in doses 600 mg/week and in combination to radiotherapy, has shown promising radiological response, partial tumor remission, prolonged median progression-free survival (PFS) and overall survival (OS), in the absence of dose-restrictive adverse effects. More robust clinical data and methodological rigor are required to further demonstrate the efficacy’s dependency on CXCL12 expression levels [4,142,143].

GLORIA and ApTOLL are pioneering CNS aptamer trials and pave the way for Ap use in human CNS therapy, although they are not strictly referring to Ap-NP conjugates.

NU 0129 provides a concrete example of clinical-stage Ap-nanoconjugate for GBM, confirming safety and CNS penetration. These trials represent foundational steps toward the clinical validation of Ap-based nanotherapeutics in neuro-oncology and neurodegeneration.

Very promising preclinical advances towards near future clinical transition in GBM are also obtained with AS1411, Gint4.T and U2 Ap, which are identified as top candidates for clinical translation thanks to their demonstrated efficacy, safety and scalable manufacturing. AS1411 Ap-NP conjugates (PTX-loaded) demonstrated enhanced drug delivery, BBB crossing, and anti-glioma effect in preclinical models; plans for toxicology and IND-enabling studies are underway [54,108,127,143]. Gint4.T and U2 Ap-polymeric NPs showed increased tumor targeting and brain uptake, radiotherapy enhancement and improved survival in GBM. The AS1411 aptamer trial (NCT00881244) is one of the few aptamer-based clinical trials, but mainly in cancer, not in neurodegenerative diseases or brain tumors [4,6,36,88,91,96,97,101,138,143].

In gliomas, the most advanced clinical trials involve ADC and exosome-based therapy, while Ap-NP conjugates are in early translational phases. Aptamer-NP-exosome conjugates delivering chemotherapeutics and gene editing CRISPR/cas-9 cargo are also in the preclinical phase with encouraging results. For instance, Depatux-M (depatuxizumab mafodotin), an ADC targeting EGFRvIII, is investigated in ongoing phase II/III trials (NCT03575886) in GBM [49,54,127,143]. Emerging exosome-based delivery of chemo- and gene therapy in gliomas is currently in early clinical trials [4,6,22,32,50,124].

In neurodegenerative disorders, ADC, aptamer-exosome conjugates and exosome delivery of siRNAs, drugs or imaging agents, are still in the preclinical phase and only exosome-related therapies have entered clinical testing. Tauopathies remain in early preclinical research stages, focusing on aptamer-based targeted delivery [4,6,8,11,30,49,53,59,61,113].

**Table 7:** Ongoing clinical trials on Ap-NP conjugates in GBM and neurodegenerative disorders.

Clinical Trial	Phase, Design and Highlights	Key Outcomes	Ref.
GLORIA	Ongoing Phase I/II multicenter, open-label, multidose escalation trial investigating the safety and efficacy of the “*L*-RNA aptamer olaptesed pegol (NOX-A12) plus radiotherapy in newly diagnosed, unresectable glioblastoma”. Primary endpoints—safety/tolerability. Status: active; approved progression into combination therapy arms (+bevacizumab; + pembrolizumab, respectively).	Strong OS (50–83% at ~15–18 months); dose-escalation: ~50%; expected to rise to ~67% as data mature. Low immunogenicity. Expansion arm (radiotherapy + bevacizumab + NOX-A12): 83% OS at median 15 months follow-up.	[4,142,143]
ApTOLL	Early phase trial investigating anti-TLR4 DNA aptamer designed to reduce inflammation and myelin loss in ischemic stroke and MS. Primary endpoints—safety and PK. Status: results under confirmation	Minimal immunogenicity Absence of serious adverse reactions First efficacy data in humans No NP-conjugate	[4,6,24,125,126]
NU-0129	Phase 0, single-arm, open-label “First in Human Study of NU 0129 (spherical nucleic acid gold nanoparticle targeting siRNA targeted to *BCL2L12* gene) in recurrent glioblastoma/gliosarcoma” Primary endpoints—safety, feasibility of i.v. administration (monitored during infusion and post-surgery) Status: Phase 0 completed. Ongoing long-term follow-up up to 2 years for survival and safety	Safe single-dose i.v. administration. Intratumoral presence of NU 0129 confirmed post-surgery. Evidence of target gene expression knockdown in surgically post-resected GBM tissue. Safe delivery without ≥grade 4 toxicities. Early PK and biomarker effect confirmed in human GBM tissue.	[4,6,10,101]

## 10. Conclusions and Future Perspectives

Aptamer nanotechnology is a very promising interdisciplinary field which can provide versatile and advantageous aptamer-nanoconjugates as specific, selective and efficient theranostics; targeted delivery carriers across the BBB and into specific brain tissues for classical drugs, antibody, siRNAs, and gene therapy; and bioimaging agents or radiosensitizers, for the major neuro-oncological and neurodegenerative disorders.

Aptamer-nanoparticle conjugates have shown encouraging preclinical safety, programmable manufacturing, and enhanced delivery profiles, especially when optimized with smart coatings and carriers. The main challenges in their clinical development are related to in vivo enzymatic and hydrolytic degradation in serum and CSF, especially of RNA aptamers; permeability through the BBB; selective and efficient brain uptake and delivery to pathological biomarkers; high rates of CSF turnover; quick renal filtration; and long-term systemic toxicity and immunogenicity. These barriers are partially solved by various strategies comprising versatile chemical modulations; multivalent surface conjugations; dual receptor-targeting aptamers; aptamers with quick delivery and high-specificity for target cells’ biomarkers; biodegradable and biocompatible already-approved nanomaterials; smart NP coatings (PEGylation, zwitterionic polymers); stimuli-responsive delivery; and exosome-based delivery. However, aptamer-nanoconjugates’ clinical application for neuro-oncological and neurodegenerative disorders still remains constrained by limited in vivo human data, unclear regulatory guidelines and the complexities concerning the validation of multicomponent theranostics.

To bridge the gap toward real-world impact and to accelerate clinical translation of promising Ap-Np conjugates as theranostics, near future research should focus on the following:

- design of aptamers based on overlapping in vivo SELEX methods with animal models, with humanized models (such as brain organoids-on-chip) and with advanced microfluidic BBB models, which could simulate more reliably and accurately the brain and BBB 3D architecture, the brain cellular diversity, or the binding dynamic to biomarkers specific to neuro-oncological and neurodegenerative pathologies [1,22,38,53,93,122];
- optimization of aptamer engineering by chemical modulations, truncated mimetic Ap and by multimerization to boost stability without compromising binding;
- protocols standardization to adopt rigorous SELEX and affinity assays within complex brain pathophysiological matrix [39,96,139];
- validation of delivery vehicles which are clinically relevant, biocompatible, scalable, reproducibly manufacturable (e.g., exosome mimetics, chemically defined conjugates), using advanced imaging and functional assays [11,24,73,75,85,93];
- clarification of molecular mechanisms downstream of aptamer engagement and incorporation of modern molecular imaging (e.g., positron emission tomography, PET, immunofluorescence) in PK/PD profiling [4,8,31,63,96,126];
- advanced PK/PD and systematic safety assessment (chronic toxicity, immunogenicity, genotoxicity, cytotoxicity) in large animal (rodents) models [6,10,59,76,139];
- comparative in vivo studies to benchmark Ap-NP conjugates against standard-of-care treatments;
- head-to-head efficacy studies to define the optimal Ap-NP platform balancing efficacy, targeting, toxicity and scale-up feasibility for reproducible manufacturing [6,38,63,143];
- regulatory harmonization, establishing frameworks tailored to nucleic acid–nanoparticle hybrids [31,69,126];
- integrated translational strategy by simultaneously addressing engineering, safety, scalability and regulatory frameworks [4,6,10,55,76,139];
- aptasensors’ translation from the lab to the clinic must further characterize their thermodynamics, target-specificity and their structure-switching profiles, as well as their integration affinity within nanoscale platforms [78,99,120,141];
- development of libraries of aptamers specifically targeting patient-specific mutations in brain tumors and tauopathies, which could be further conjugated to NPs and medicines, in order to guide personalized diagnosis and therapy in neuro-oncological and neurodegenerative disorders [4,6,51,139,143].

## Data Availability

Not applicable.

## Abbreviations

The following abbreviations are used in this manuscript:

AD	Alzheimer’s disease
ADC	Antibody-drug conjugate
ADNFs	Aptamer-bound DNA nanoflowers
Ap	Aptamers
Ap-NP	Aptamer-nanoparticle conjugates
ATP	Adenosine triphosphate
Aβ	Amyloid β-protein
BBB	Blood–brain barrier
BDNF	Brain-derived neurotrophic factor
chemo-PDT	Chemo-photodynamic therapy
circRNA	circular RNA
CPT	Camptothecin
CSF	Cerebrospinal fluid
CTLA-4	Cytotoxic T-lymphocyte-associated protein 4
CXCL12	Stromal cell-derived factor-1
CXCR4	C-X-C Motif chemokine receptor 4
DOX	Doxorubicin
DTX	Docetaxel
EAE	Autoimmune encephalomyelitis
EGFR	Epidermal growth factor receptor
ELISA	Enzyme-linked immunosorbent assay
EpCAM	Epithelial cell adhesion molecule
EPR	Enhanced permeability and retention
GBM	Glioblastoma multiforme
Gd	Gadolinium
GFP	Green fluorescent protein
GMP	Good manufacturing practice
gRNA	Gene-editing guide RNA
FDA	Food and drug administration
HAT1	Histone acetyl transferase-1
HTS	High-throughput screening
idT	inverted (deoxy-) thymidine
LM	Leptomeningeal carcinomatosis
i.p.; i.v.; i.c.v.	Intraperitoneal; intravenous; intracerebroventricular
mAb	Monoclonal antibodies
miRNA	microRNA
MS	Multiple sclerosis
NA	Nucleic acid
NCL	Nucleolin
NDs	Neurodegenerative diseases
oAβ	Oligomeric amyloid beta
OGN	Oligonucleotide
OPC	oligodendrocyte progenitor cell
OS	Overall survival
PCR	Polymerase chain reaction
PAMAM	Hydroxyl-terminated poly(amidoamine) dendrimer
PK	Pharmacokinetics
PK/PD	Pharmacokinetics/pharmacodynamics profile
PD	Parkinson’s disease
PD-1	Programmed cell death 1
PDGFRβ	Human platelet-derived growth factor receptor
PD-L1	Programmed cell death ligand 1
PDT	Photodynamic therapy
PEG	Polyethylene glycol
PEG-PCL	PEG-poly(ε-caprolactone)
PET	Positron emission tomography
PFS	Progression free survival
PGG-PTX	(*L-γ*- Glutamyl-glutamine)-paclitaxel
PLGA-b-PEG	Poly(lactico-glycolic)-block-polyethylene glycol
PrPC	Cellular prionic form
PrPSc	Prionic infectious proteins (scrapie form)
PSMA	Prostate specific membrane antigen
QD	Quantum dot
Ref.	References
RES	Reticuloendothelial system
RGD	Arginine-glycine-aspartate
RVG	Rabies virus glycoprotein peptide
SELEX	Systematic Evolution of ligands by exponential enrichment
siHDGF	Small interfering RNA hepatoma-derived growth factor
siRNA	Small interfering RNA
αSyn	alpha-Synuclein
ssDNA, ssRNA	Single-stranded DNA or RNA
Tau τ	Protein Tau
TfR	Transferrin receptor
TGF	Transforming growth factor
TLR4	Toll-like receptor 4
TGN	a peptide of 12 amino acids TGNYKALHPHN
TME	Tumor microenvironment
TMZ	Temozolomide
TrkB	Tropomyosin receptor kinase B
UCNP	Upconversion nanoparticles
VEGF	Vascular endothelial growth factor
VitC	Vitamin C

## References

[1] Koshmanova A.A., Artyushenko P.V., Shchugoreva I.A., Fedotovskaya V.D., Luzan N.A., Kolovskaya O.S., Zamay G.S., Lukyanenko K.A., Veprintsev D.V., Khilazheva E.D. (2024). Aptamer’s Structure Optimization for Better Diagnosis and Treatment of Glial Tumors. Cancers.

[2] Yu A.-M., Tu M.-J. (2022). Deliver the promise: RNAs as a new class of molecular entities for therapy and vaccination. Pharmacol. Ther..

[3] Guan B., Zhang X. (2020). Aptamers as Versatile Ligands for Biomedical and Pharmaceutical Applications. Int. J. Nanomed..

[4] Cesarini V., Appleton S.L., De Franciscis V., Catalucci D. (2025). The recent blooming of therapeutic aptamers. Mol. Aspects Med..

[5] Suelves A.M., Shulman J.P. (2017). Anti-Vascular Endothelial Growth Factor Medications in Retinopathy of Prematurity. Adv. Ophthalmol. Optom..

[6] Bege M., Ghanem K.R., Borbás A. (2025). The 20th Anniversary of Pegaptanib (MacugenTM), the First Approved Aptamer Medicine: History, Recent Advances and Future Prospects of Aptamers in Therapy. Pharmaceutics..

[7] Mullard A. (2023). FDA approves second RNA aptamer. Nat. Rev. Drug Discov..

[8] Wang B., Kobeissy F., Golpich M., Cai G., Li X., Abedi R., Haskins W., Tan W., Benner S.A., Wang K.K.W. (2024). Aptamer Technologies in Neuroscience, Neuro-Diagnostics and Neuro-Medicine Development. Molecules.

[9] Tian L., Qi J., Qian K., Oderinde O., Liu Q., Yao C., Song W., Wang Y. (2018). Copper (II) oxide nanozyme based electrochemical cytosensor for high sensitive detection of circulating tumor cells in breast cancer. J. Electroanal. Chem..

[10] Doherty C., Wilbanks B., Khatua S., Maher L.J. (2024). Aptamers in neuro-oncology: An emerging therapeutic modality. Neuro-Oncology.

[11] Song B., Wang X., Qin L., Hussain S., Liang W. (2024). Brain gliomas: Diagnostic and therapeutic issues and the prospects of drug-targeted nano-delivery technology. Pharmacol. Res..

[12] Delač M., Motaln H., Ulrich H., Lah T.T. (2015). Aptamer for imaging and therapeutic targeting of brain tumor glioblastoma. Cytometry A.

[13] Giles B., Nakhjavani M., Wiesa A., Knight T., Shigdar S., Samarasinghe R.M. (2023). Unravelling the Glioblastoma Tumour Microenvironment: Can Aptamer Targeted Delivery Become Successful in Treating Brain Cancers?. Cancers.

[14] Bellelli F., Angioni D., Arosio B., Vellas B., De Souto Barreto P. (2025). Hallmarks of Aging and Alzheimer’s Disease Pathogenesis: Paving the Route for New Therapeutic Targets. Ageing Res. Rev..

[15] Nimjee S.M., White R.R., Becker R.C., Sullenger B.A. (2017). Aptamers as Therapeutics. Annu. Rev. Pharmacol. Toxicol..

[16] Nimjee S.M., Sullenger B.A. (2020). Therapeutic Aptamers: Evolving to Find their Clinical Niche. Curr. Med. Chem..

[17] Sajid M.I., Moazzam M., Kato S., Cho K.Y., Tiwari R.K. (2020). Overcoming Barriers for siRNA Therapeutics: From Bench to Bedside. Pharmaceuticals.

[18] Zhou J., Rossi J.J. (2011). Cell-Specific Aptamer-Mediated Targeted Drug Delivery. Oligonucleotides.

[19] Barrera-Ocampo A. (2024). Monoclonal Antibodies and Aptamers: The Future Therapeutics for Alzheimer’s Disease. Acta Pharm. Sin. B.

[20] Murakami K., Izuo N., Bitan G. (2022). Aptamers targeting amyloidogenic proteins and their emerging role in neurodegenerative diseases. J. Biol. Chem..

[21] Shraim A.S., Abdel Majeed B.A., Al-Binni M.A., Hunaiti A. (2022). Therapeutic Potential of Aptamer–Protein Interactions. ACS Pharmacol. Transl. Sci..

[22] Wang B., Pan X., Teng I., Li X., Kobeissy F., Wu Z., Zhu J., Cai G., Yan H., Yan X. (2024). Functional Selection of Tau Oligomerization–Inhibiting Aptamers. Angew. Chem. Int. Ed..

[23] Chatterjee B., Das S.J., Anand A., Sharma T.K. (2020). Nanozymes and aptamer-based biosensing. Mater. Sci. Energy Technol..

[24] Moreira R., Nóbrega C., De Almeida L.P., Mendonça L. (2024). Brain-targeted drug delivery—Nanovesicles directed to specific brain cells by brain-targeting ligands. J. Nanobiotechnol..

[25] Jeevanandam J., Tan K.X., Danquah M.K., Guo H., Turgeson A. (2020). Advancing Aptamers as Molecular Probes for Cancer Theranostic Applications-The Role of Molecular Dynamics Simulation. Biotechnol. J..

[26] Duan Q., Jia H., Chen W., Qin C., Zhang K., Jia F., Fu T., Wei Y., Fan M., Wu Q. (2024). Multivalent Aptamer-Based Lysosome-Targeting Chimeras (LYTACs) Platform for Mono or Dual Targeted Proteins Degradation on Cell Surface. Adv. Sci..

[27] Sonali Viswanadh M.K., Singh R.P., Agrawal P., Mehata A.K., Pawde D.M., Narendra Sonkar R., Muthu M.S. (2018). Nanotheranostics: Emerging Strategies for Early Diagnosis and Therapy of Brain Cancer. Nanotheranostics.

[28] Xie S., Sun W., Fu T., Liu X., Chen P., Qiu L., Qu F., Tan W. (2023). Aptamer-Based Targeted Delivery of Functional Nucleic Acids. J. Am. Chem. Soc..

[29] Liu M., Wang L., Lo Y., Shiu S.C.-C., Kinghorn A.B., Tanner J.A. (2022). Aptamer-Enabled Nanomaterials for Therapeutics, Drug Targeting and Imaging. Cells.

[30] Kim H.J., Sung H.J., Lee Y.M., Choi S.I., Kim Y.H., Heo K., Kim I.H. (2020). Therapeutic Application of Drug-Conjugated HER2 Oligobody (HER2-DOligobody). Int. J. Mol. Sci..

[31] Shoemaker R.L., Larsen R.J., Larsen P.A. (2024). Single-Domain Antibodies and Aptamers Drive New Opportunities for Neurodegenerative Disease Research. Front. Immunol..

[32] Salarpour S., Barani M., Pardakhty A., Khatami M., Pal Singh Chauhan N. (2022). The application of exosomes and Exosome-nanoparticle in treating brain disorders. J. Mol. Liq..

[33] Liang S., Xu H., Ye B.-C. (2022). Membrane-Decorated Exosomes for Combination Drug Delivery and Improved Glioma Therapy. Langmuir.

[34] Shamili F.H., Alibolandi M., Rafatpanah H., Abnous K., Mahmoudi M., Kalantari M., Taghdisi S.M., Ramezani M. (2019). Immunomodulatory properties of MSC-derived exosomes armed with high affinity aptamer toward myelin as a platform for reducing multiple sclerosis clinical score. J. Control. Release.

[35] Kozma G.T., Shimizu T., Ishida T., Szebeni J. (2020). Anti-PEG antibodies: Properties, formation, testing and role in adverse immune reactions to PEGylated nano-biopharmaceuticals. Adv. Drug Deliv. Rev..

[36] Rabiee N., Chen S., Ahmadi S., Veedu R.N. (2023). Aptamer-engineered (nano)materials for theranostic applications. Theranostics.

[37] Herrera A., Zhou J., Song M., Rossi J.J., Lin R.-J. (2023). Evolution of Cell-Type-Specific RNA Aptamers via Live Cell-Based SELEX. RNA-Protein Complexes and Interactions.

[38] Meehan C., Lecocq S., Penner G. (2024). A reproducible approach for the use of aptamer libraries for the identification of Aptamarkers for brain amyloid deposition based on plasma analysis. PLoS ONE.

[39] Didarian R., Ozbek H.K., Ozalp V.C., Erel O., Nimet Yildirim-Tirgil N. (2024). Enhanced SELEX Platforms for Aptamer Selection with Improved Characteristics: A Review. Mol. Biotechnol..

[40] Egli M., Manoharan M. (2023). Chemistry, structure and function of approved oligonucleotide therapeutics. Nucleic Acids Res..

[41] Shigdar S., Agnello L., Fedele M., Camorani S., Cerchia L. (2021). Profiling Cancer Cells by Cell-SELEX: Use of Aptamers for Discovery of Actionable Biomarkers and Therapeutic Applications Thereof. Pharmaceutics.

[42] Sellés Vidal L., Isalan M., Heap J.T., Ledesma-Amaro R. (2023). A primer to directed evolution: Current methodologies and future directions. RSC Chem. Biol..

[43] Rtools. http://rtools.cbrc.jp/.

[44] Buglak A.A., Samokhvalov A.V., Zherdev A.V., Dzantiev B.B. (2020). Methods and Applications of In Silico Aptamer Design and Modeling. Int. J. Mol. Sci..

[45] Joyce T., Tasci E., Jagasia S., Shephard J., Chappidi S., Zhuge Y., Zhang L., Cooley Zgela T., Sproull M., Mackey M. (2024). Serum CD133-Associated Proteins Identified by Machine Learning Are Connected to Neural Development, Cancer Pathways, and 12-Month Survival in Glioblastoma. Cancers.

[46] Dasti A., Cid-Samper F., Bechara E., Tartaglia G.G. (2020). RNA-centric approaches to study RNA-protein interactions in vitro and in silico. Methods.

[47] He S., Du Y., Tao H., Duan H. (2023). Advances in aptamer-mediated targeted delivery system for cancer treatment. Int. J. Biol. Macromol..

[48] Wong K.-Y., Wong M.-S., Liu J. (2024). Aptamer-functionalized liposomes for drug delivery. Biomed. J..

[49] Furtado D., Björnmalm M., Ayton S., Bush A.I., Kempe K., Caruso F. (2018). Overcoming the Blood-Brain Barrier: The Role of Nanomaterials in Treating Neurological Diseases. Adv. Mater..

[50] Amero P., Khatua S., Rodriguez-Aguayo C., Lopez-Berestein G. (2020). Aptamers: Novel Therapeutics and Potential Role in Neuro-Oncology. Cancers.

[51] Ismail M., Liu J., Wang N., Zhang D., Qin C., Shi B., Zheng M. (2025). Advanced nanoparticle engineering for precision therapeutics of brain diseases. Biomaterials.

[52] Khongkow M., Yata T., Boonrungsiman S., Ruktanonchai U.R., Graham D., Namdee K. (2019). Surface modification of gold nanoparticles with neuron-targeted exosome for enhanced blood-brain barrier penetration. Sci. Rep..

[53] Rafati N., Zarepour A., Bigham A., Khosravi A., Naderi-Manesh H., Iravani S., Zarrabi A. (2024). Nanosystems for targeted drug Delivery: Innovations and challenges in overcoming the Blood-Brain barrier for neurodegenerative disease and cancer therapy. Int. J. Pharm..

[54] Ghaffari M., Sanadgol N., Abdollahi M. (2020). A Systematic Review of Current Progresses in the Nucleic Acid-Based Therapies for Neurodegeneration with Implications for Alzheimer’s Disease. Mini Rev. Med. Chem..

[55] Urmi R., Banerjee P., Singh M., Singh R., Chhillar S., Sharma N., Chandra A., Singh N., Qamar I. (2024). Revolutionizing biomedicine: Aptamer-based nanomaterials and nanodevices for therapeutic applications. Biotechnol. Rep..

[56] Xie S., Du Y., Zhang Y., Wang Z., Zhang D., He L., Qiu L., Jiang J., Tan W. (2020). Aptamer-based optical manipulation of protein subcellular localization in cells. Nat. Commun..

[57] Li Y., Liu R., Zhao Z. (2025). Targeting Brain Drug Delivery with Macromolecules Through Receptor-Mediated Transcytosis. Pharmaceutics.

[58] Mi P., Cabral H., Kataoka K. (2020). Ligand-Installed Nanocarriers toward Precision Therapy. Adv. Mater..

[59] Mahmoudian F., Ahmari A., Shabani S., Sadeghi B., Fahimirad S., Fattahi F. (2024). Aptamers as an Approach to Targeted Cancer Therapy. Cancer Cell Int..

[60] Ni S., Yao H., Wang L., Lu J., Jiang F., Lu A., Zhang G. (2017). Chemical Modifications of Nucleic Acid Aptamers for Therapeutic Purposes. Int. J. Mol. Sci..

[61] Byun J. (2021). Recent Progress and Opportunities for Nucleic Acid Aptamers. Life.

[62] Duro-Castano A., Borrás C., Herranz-Pérez V., Blanco-Gandía M.C., Conejos-Sánchez I., Armiñán A., Mas-Bargues C., Inglés M., Miñarro J., Rodríguez-Arias M. (2021). Targeting Alzheimer’s disease with multimodal polypeptide-based nanoconjugates. Sci. Adv..

[63] Garima, Imtiyaz K., Pooja, Pannu P., Sharma A., Raina S., Kumar S., Anwer S.T., Alam Rizvi M., Sinha S.K., Barkat M.A., Ahmad F.J., Rahman M.A., Ansari M.A. (2024). Theranostic Application of Nanomedicine in Neurodegenerative Diseases: Current and Future Perspectives. Nanotheranostics for Diagnosis and Therapy.

[64] Aggarwal N., Choudhury S., Chibh S., Panda J.J. (2022). Aptamer-nanoconjugates as emerging theranostic systems in neurodegenerative disorders. Colloid. Interface Sci. Commun..

[65] Zhong C., Shi Z., Binzel D.W., Jin K., Li X., Guo P., Li S.K. (2024). Posterior eye delivery of angiogenesis-inhibiting RNA nanoparticles via subconjunctival injection. Int. J. Pharm..

[66] Ebenezer O., Comoglio P., Wong G.K.-S., Tuszynski J.A. (2023). Development of Novel siRNA Therapeutics: A Review with a Focus on Inclisiran for the Treatment of Hypercholesterolemia. Int. J. Mol. Sci..

[67] Janssen M., Stenmark H., Carlson A. (2021). Divalent ligand-monovalent molecule binding. Soft Matter.

[68] Yang C., Zhao H., Sun Y., Wang C., Geng X., Wang R., Tang L., Han D., Liu J., Tan W. (2022). Programmable manipulation of oligonucleotide-albumin interaction for elongated circulation time. Nucleic Acids Res..

[69] Di Mauro V., Lauta F.C., Modica J., Appleton S.L., De Franciscis V., Catalucci D. (2024). Diagnostic and Therapeutic Aptamers. JACC Basic. Transl. Sci..

[70] Al Mazid M.F., Shkel O., Ryu E., Kim J., Shin K.H., Kim Y.K., Lim H.S., Lee J.-S. (2024). Aptamer and N-Degron Ensemble (AptaGron) as a Target Protein Degradation Strategy. ACS Chem. Biol..

[71] Chen B.-M., Cheng T.-L., Roffler S.R. (2021). Polyethylene Glycol Immunogenicity: Theoretical, Clinical, and Practical Aspects of Anti-Polyethylene Glycol Antibodies. ACS Nano.

[72] Sun C.Y., Cao Z., Zhang X.J., Sun R., Yu C.S., Yang X. (2018). Cascade-amplifying synergistic effects of chemo-photodynamic therapy using ROS-responsive polymeric nanocarriers. Theranostics.

[73] Driscoll J., Gondaliya P., Zinn D.A., Jain R., Yan I.K., Dong H., Patel T. (2025). Using aptamers for targeted delivery of RNA therapies. Mol. Ther..

[74] Pérez-López A., Torres-Suárez A.I., Martín-Sabroso C., Aparicio-Blanco J. (2023). An overview of in vitro 3D models of the blood-brain barrier as a tool to predict the in vivo permeability of nanomedicines. Adv. Drug Deliv. Rev..

[75] Culkins C., Adomanis R., Phan N., Robinson B., Slaton E., Lothrop E., Chen Y., Kimmel B.R. (2024). Unlocking the Gates: Therapeutic Agents for Noninvasive Drug Delivery Across the Blood-Brain Barrier. Mol. Pharm..

[76] Dume B., Licarete E., Banciu M. (2024). Advancing cancer treatments: The role of oligonucleotide-based therapies in driving progress. Mol. Ther. Nucleic Acids.

[77] Belfiore L., Saunders D.N., Ranson M., Thurecht K.J., Storm G., Vine K.L. (2018). Towards clinical translation of ligand-functionalized liposomes in targeted cancer therapy: Challenges and opportunities. J. Control. Release.

[78] Stuber A., Nakatsuka N. (2024). Aptamer Renaissance for Neurochemical Biosensing. ACS Nano.

[79] Shirmast P., Shahri M.A., Brent A., Idris A., McMillan N.A.J. (2024). Delivering therapeutic RNA into the brain using extracellular vesicles. Mol. Ther. Nucleic Acids.

[80] Choi J.-W., Seo M., Kim K., Kim A.-R., Lee H., Kim H.-S., Park C.G., Cho S.W., Kang J.H., Joo J. (2023). Aptamer Nanoconstructs Crossing Human Blood-Brain Barrier Discovered via Microphysiological System-Based SELEX Technology. ACS Nano.

[81] Macdonald J., Denoyer D., Henri J., Jamieson A., Burvenich I.J.G., Pouliot N., Shigdar S. (2020). Bifunctional Aptamer-Doxorubicin Conjugate Crosses the Blood-Brain Barrier and Selectively Delivers Its Payload to EpCAM-Positive Tumor Cells. Nucleic Acid. Ther..

[82] Ozturk M., Nilsen-Hamilton M., Ilgu M. (2021). Aptamer Applications in Neuroscience. Pharmaceuticals.

[83] Ghasemii K., Darroudi M., Rahimmanesh I., Ghomi M., Hassanpour M., Sharifi E., Yousefiasl S., Ahmadi S., Zarrabi A., Borzacchiello A. (2022). Advances in aptamer-based drug delivery vehicles for cancer therapy. Biomater. Adv..

[84] Brylev V.A., Ryabukhina E.V., Nazarova E.V., Samoylenkova N.S., Gulyak E.L., Sapozhnikova K.A., Dzarieva F.M., Ustinov A.V., Pronin I.N., Usachev D.Y. (2024). Towards Aptamer-Targeted Drug Delivery to Brain Tumors: The Synthesis of Ramified Conjugates of an EGFR-Specific Aptamer with MMAE on a Cathepsin B-Cleavable Linker. Pharmaceutics.

[85] Hang Z., Zhou L., Bian X., Liu G., Cui F., Du H., Wen Y. (2024). Potential application of aptamers combined with DNA nanoflowers in neurodegenerative diseases. Ageing Res. Rev..

[86] Mishra A., Kumar R., Mishra J., Dutta K., Ahlawat P., Kumar A., Dhanasekaran S., Gupta A.K., Sinha S., Bishi D.K. (2023). Strategies facilitating the permeation of nanoparticles through blood-brain barrier: An insight towards the development of brain-targeted drug delivery system. J. Drug Deliv. Sci. Technol..

[87] Mitchell M.J., Billingsley M.M., Haley R.M., Wechsler M.E., Peppas N.A., Langer R. (2021). Engineering precision nanoparticles for drug delivery. Nat. Rev. Drug Discov..

[88] Monaco I., Camorani S., Colecchia D., Locatelli E., Calandro P., Oudin A., Niclou S., Arra C., Chiariello M., Cerchia L. (2017). Aptamer Functionalization of Nanosystems for Glioblastoma Targeting through the Blood-Brain Barrier. J. Med. Chem..

[89] Gil-Cabrerizo P., Simon-Yarza T., Garbayo E., Blanco-Prieto M.J. (2024). Navigating the landscape of RNA delivery systems in cardiovascular disease therapeutics. Adv. Drug Deliv. Rev..

[90] Pastor F., Soldevilla M.M., Villanueva H., Kolonias D., Inoges S., De Cerio A.L., Kandzia R., Klimyuk V., Gleba Y., Gilboa E. (2013). CD28 Aptamers as Powerful Immune Response Modulators. Mol. Ther. Nucleic Acids.

[91] Yadav P., Ambudkar S.V., Rajendra Prasad N. (2022). Emerging nanotechnology-based therapeutics to combat multidrug-resistant cancer. J. Nanobiotechnol..

[92] Thomas B.J., Guldenpfennig C., Guan Y., Winkler C., Beecher M., Beedy M., Berendzen A.F., Ma L., Daniels M.A., Burke D.H. (2023). Targeting lung cancer with clinically relevant EGFR mutations using anti-EGFR RNA aptamer. Mol. Ther. Nucleic Acids.

[93] Chowdhury R., Eslami S., Pham C.V., Rai A., Lin J., Hou Y., Greening D.W., Duan W. (2024). Role of aptamer technology in extracellular vesicle biology and therapeutic applications. Nanoscale.

[94] Booth B.J., Nourreddine S., Katrekar D., Savva Y., Bose D., Long T.J., Huss D.J., Mali P. (2023). RNA editing: Expanding the potential of RNA therapeutics. Mol. Ther..

[95] Zhang M.M., Bahal R., Rasmussen T.P., Manautou J.E., Zhong X. (2021). The growth of siRNA-based therapeutics: Updated clinical studies. Biochem. Pharmacol..

[96] Seo K., Hwang K., Nam K.M., Kim M.J., Song Y.-K., Kim C.-Y. (2024). Nucleolin-Targeting AS1411 Aptamer-Conjugated Nanospheres for Targeted Treatment of Glioblastoma. Pharmaceutics.

[97] Luo Z., Yan Z., Jin K., Pang Q., Jiang T., Lu H., Liu X., Pang Z., Yu L., Jiang X. (2017). Precise Glioblastoma Targeting by AS1411 Aptamer-Functionalized Poly(L-γ-glutamylglutamine)-Paclitaxel Nanoconjugates. J. Colloid. Interface Sci..

[98] Chauhan M., Singh R.P., Sonali, Yadav B., Shekhar S., Kumar L., Mehata A.K., Jhawat V., Dutt R., Garg V. (2023). Dual-Targeted Transferrin and AS1411 Aptamer Conjugated Micelles for Improved Therapeutic Efficacy and Imaging of Brain Cancer. Colloids Surf. B Biointerfaces.

[99] Chauhan M., Sonali, Shekhar S., Yadav B., Garg V., Dutt R., Mehata A.K., Goswami P., Koch B., Muthu M.S. (2024). AS1411 Aptamer/RGD Dual Functionalized Theranostic Chitosan-PLGA Nanoparticles for Brain Cancer Treatment and Imaging. Biomater. Adv..

[100] Fei H., Jin Y., Jiang N., Zhou Y., Wei N., Liu Y., Miao J., Zhang L., Li R., Zhang A. (2024). Gint4.T-siHDGF Chimera-Capped Mesoporous Silica Nanoparticles Encapsulating Temozolomide for Synergistic Glioblastoma Therapy. Biomaterials.

[101] Esposito C.L., Nuzzo S., Catuogno S., Romano S., De Nigris F., De Franciscis V. (2018). STAT3 Gene Silencing by Aptamer-siRNA Chimera as Selective Therapeutic for Glioblastoma. Mol. Ther. Nucleic Acids.

[102] Yoon S., Huang K.-W., Andrikakou P., Vasconcelos D., Swiderski P., Reebye V., Sodergren M., Habib N., Rossi J.J. (2019). Targeted Delivery of C/EBPα-saRNA by RNA Aptamers Shows Anti-Tumor Effects in a Mouse Model of Advanced PDAC. Mol. Ther. Nucleic Acids.

[103] Huang B.-T., Lai W.-Y., Yeh C.-L., Tseng Y.-T., Peck K., Yang P.-C., Lin E.P.-Y. (2024). AptBCis1, an Aptamer-Cisplatin Conjugate, Is Effective in Lung Cancer Leptomeningeal Carcinomatosis. ACS Nano.

[104] Nuzzo S., Brancato V., Affinito A., Salvatore M., Cavaliere C., Condorelli G. (2020). The Role of RNA and DNA Aptamers in Glioblastoma Diagnosis and Therapy: A Systematic Review of the Literature. Cancers.

[105] Liyanage W., Wu T., Kannan S., Kannan R.M. (2022). Dendrimer-siRNA Conjugates for Targeted Intracellular Delivery in Glioblastoma Animal Models. ACS Appl. Mater. Interfaces..

[106] Tang J., Huang N., Zhang X., Zhou T., Tan Y., Pi J., Pi L., Cheng S., Zheng H., Cheng Y. (2017). Aptamer-conjugated PEGylated quantum dots targeting epidermal growth factor receptor variant III for fluorescence imaging of glioma. Int. J. Nanomed..

[107] Wei J., Song R., Sabbagh A., Marisetty A., Shukla N., Fang D., Najem H., Ott M., Long J., Zhai L. (2022). Cell-Directed Aptamer Therapeutic Targeting for Cancers Including Those within the Central Nervous System. OncoImmunology.

[108] Bar-Zeev M., Livney Y.D., Assaraf Y.G. (2017). Targeted nanomedicine for cancer therapeutics: Towards precision medicine overcoming drug resistance. Drug Resist. Updat..

[109] Xu T., Wang H., Huang X., Li W., Huang Q., Yan Y., Chen J. (2018). Gene Fusion in Malignant Glioma: An Emerging Target for Next-Generation Personalized Treatment. Transl. Oncol..

[110] Jain N., Zhu H., Khashab T., Ye Q., George B., Mathur R., Singh R.K., Berkova Z., Wise J.F., Braun F.K. (2018). Targeting Nucleolin for Better Survival in Diffuse Large B-Cell Lymphoma. Leukemia.

[111] Pavlova S., Fab L., Dzarieva F., Ryabova A., Revishchin A., Panteleev D., Antipova O., Usachev D., Kopylov A., Pavlova G. (2024). Unite and Conquer: Association of Two G-Quadruplex Aptamers Provides Antiproliferative and Antimigration Activity for Cells from High-Grade Glioma Patients. Pharmaceuticals.

[112] Ausejo-Mauleon I., Martinez-Velez N., Lacalle A., De La Nava D., Cebollero J., Villanueva H., Casares N., Marco-Sanz J., Laspidea V., Becher O. (2024). Combination of Locoregional Radio-Therapy with a TIM-3 Aptamer Improves Survival in Diffuse Midline Glioma Models. JCI Insight.

[113] Maimaitiyiming Y., Hong F., Yang C., Naranmandura H. (2019). Novel insights into the role of aptamers in the fight against cancer. J. Cancer Res. Clin. Oncol..

[114] Ko H.Y., Choi K.-J., Lee C.H., Kim S. (2011). A multimodal nanoparticle-based cancer imaging probe simultaneously targeting nucleolin, integrin αvβ3 and tenascin-C proteins. Biomaterials.

[115] Hwang D.W., Ko H.Y., Lee J.H., Kang H., Ryu S.H., Song I.C., Lee D.S., Kim S. (2010). A Nucleolin-Targeted Multimodal Nanoparticle Imaging Probe for Tracking Cancer Cells Using an Aptamer. J. Nucl. Med..

[116] Ju I.G., Lee J.H., Lee J.-M., Im H., Eo H., Moon M., Song M.K., Kim Y.-S., Oh M.S., Kim Y.-J. (2025). NXP031 Restores Memory Function by Dual Effects Degrading Aβ Accumulation and Facilitating Antioxidant Pathway in Alzheimer’s Disease Models. Free Radic. Biol. Med..

[117] Kan X., Ma J., Ma J., Li D., Li F., Cao Y., Huang C., Li Y., Liu P. (2025). Dual-Targeted TfRA4-DNA1-Ag@AuNPs: An Innovative Radiosensitizer for Enhancing Radiotherapy in Glioblastoma Multiforme. Colloids Surf. B Biointerfaces.

[118] Li D., Zhao J., Ma J., Yang H., Zhang X., Cao Y., Liu P. (2022). GMT8 Aptamer Conjugated PEGylated Ag@Au Core-Shell Nanoparticles as a Novel Radiosensitizer for Targeted Radiotherapy of Glioma. Colloids Surf. B Biointerfaces.

[119] Teng I.-T., Li X., Yadikar H.A., Yang Z., Li L., Lyu Y., Pan X., Wang K.K., Tan W. (2018). Identification and Characterization of DNA Aptamers Specific for Phosphorylation Epitopes of Tau Protein. J. Am. Chem. Soc..

[120] Jia Z., Maghaydah Y., Zdanys K., Kuchel G.A., Diniz B.S., Liu C. (2024). CRISPR-Powered Aptasensor for Diagnostics of Alzheimer’s Disease. ACS Sens..

[121] Dar K.B., Bhat A.H., Amin S., Reshi B.A., Zargar M.A., Masood A., Ganie S.A. (2020). Elucidating Critical Proteinopathic Mechanisms and Potential Drug Targets in Neurodegeneration. Cell. Mol. Neurobiol..

[122] McConnell E.M., Chan D., Ventura K., Callahan J.P., Harris K., Hunt V.H., Boisjoli S., Knight D., Monk E.T., Holahan M.R. (2024). Selection of DNA Aptamers That Prevent the Fibrillization of α-Synuclein Protein in Cellular and Mouse Models. Mol. Ther. Nucleic Acids.

[123] Sensi S.L., Russo M., Tiraboschi P. (2023). Biomarkers of diagnosis, prognosis, pathogenesis, response to therapy: Convergence or divergence? Lessons from Alzheimer’s disease and synucleinopathies. Handb. Clin. Neurol..

[124] Pichla M., Bartosz G., Sadowska-Bartosz I. (2020). The Antiaggregative and Antiamyloidogenic Properties of Nanoparticles: A Promising Tool for the Treatment and Diagnostics of Neurodegenerative Diseases. Oxid. Med. Cell. Longev..

[125] Fernández-Gómez B., Marchena M.A., Piñeiro D., Gómez-Martín P., Sánchez E., Laó Y., Valencia G., Nocera S., Benítez-Fernández R., Castaño-León A.M. (2024). ApTOLL: A new therapeutic aptamer for cytoprotection and (re)myelination after multiple sclerosis. Br. J. Pharmacol..

[126] Aliena-Valero A., Hernández-Jiménez M., López-Morales M.A., Tamayo-Torres E., Castelló-Ruiz M., Piñeiro D., Ribó M., Salom J.B. (2024). Cerebroprotective Effects of the TLR4-Binding DNA Aptamer ApTOLL in a Rat Model of Ischemic Stroke and Thrombectomy Recanalization. Pharmaceutics.

[127] Gote V., Nookala A.R., Bolla P.K., Pal D. (2021). Drug Resistance in Metastatic Breast Cancer: Tumor Targeted Nanomedicine to the Rescue. Int. J. Mol. Sci..

[128] Chowdhury A., Collins J.M., Gell D.A., Perry S., Breadmore M.C., Shigdar S., King A.E. (2024). Isolation and Identification of the High-Affinity DNA Aptamer Target to the Brain-Derived Neurotrophic Factor (BDNF). ACS Chem. Neurosci..

[129] Shan L. (2022). Indotricarbocyanine-loaded AS1411 DNA aptamer- and TGN peptide-modified poly(ethylene glycol)-poly(ε-caprolactone) nanoparticles. Molecular Imaging and Contrast Agent Database (MICAD).

[130] Fechter P., Da Silva E.C., Mercier M.-C., Noulet F., Etienne-Seloum N., Guenot D., Lehmann M., Vauchelles R., Martin S., Lelong-Rebel I. (2019). RNA Aptamers Targeting Integrin α5β1 as Probes for Cyto- and Histofluorescence in Glioblastoma. Mol. Ther. Nucleic Acids.

[131] Banik M., Ledray A.P., Wu Y., Lu Y. (2024). Delivering DNA Aptamers Across the Blood-Brain Barrier Reveals Heterogeneous Decreased ATP in Different Brain Regions of Alzheimer’s Disease Mouse Models. ACS Cent. Sci..

[132] Wu L., Zhang Y., Wang Z., Zhang Y., Zou J., Qiu L. (2022). Aptamer-Based Cancer Cell Analysis and Treatment. ChemistryOpen.

[133] Mikula E., Malecka-Baturo K. (2023). An Overview of the Latest Developments in the Electrochemical Aptasensing of Neurodegenerative Diseases. Coatings.

[134] Walter H.-K., Bauer J., Steinmeyer J., Kuzuya A., Niemeyer C.M., Wagenknecht H.-A. (2017). “DNA Origami Traffic Lights” with a Split Aptamer Sensor for a Bicolor Fluorescence Readout. Nano Lett..

[135] Bargiela-Cuevas S., Marin M., Gabaldon-Ojeda M., Klett-Mingo J.I., Granado P., Sacristan S., Esteban-Lasso A., Casas J.G., Martin M.E., González V.M.M. (2024). Histone Acetyl Transferase 1 Is Overexpressed in Poor Prognosis, High-grade Meningeal and Glial Brain Cancers: Immunohistochemical and Aptahistochemical Study. J. Histochem. Cytochem..

[136] Iyer A.K., Singh A., Ganta S., Amiji M.M. (2013). Role of integrated cancer nanomedicine in overcoming drug resistance. Adv. Drug Deliv. Rev..

[137] Salehirozveh M., Bonné R., Kumar P., Abazar F., Dehghani P., Mijakovic I., Roy V.A.L. (2025). Enhanced Detection of Brain-Derived Neurotrophic Factor (BDNF) Using a Reduced Graphene Oxide Field-Effect Transistor Aptasensor. Nanoscale.

[138] Khot V.M., Salunkhe A.B., Pricl S., Bauer J., Thorat N.D., Townley H. (2021). Nanomedicine-driven molecular targeting, drug delivery, and therapeutic approaches to cancer chemoresistance. Drug Discov. Today.

[139] Muhammad M., Liu C., Yang G., Shao C.-S., Xiong L., Xia H., Iqbal J., Zhan J., Qu F., Huang Q. (2025). Early-stage Alzheimer’s disease profiling in blood achieved by multiplexing aptamer-SERS biosensors. Biosens. Bioelectron..

[140] Bodily T.A., Ramanathan A., Wei S., Karkisaval A., Bhatt N., Jerez C., Haque A., Ramil A., Heda P., Wang Y. (2023). In pursuit of degenerative brain disease diagnosis: Dementia biomarkers detected by DNA aptamer-attached portable graphene biosensor. Proc. Natl. Acad. Sci. USA.

[141] Park J.W., Tian Y., Kim S.-T., Park C., Kim Y.M., Chung H.K., Kim K.M., Jahng G.-H. (2024). Oligomeric amyloid-β targeted contrast agent for MRI evaluation of Alzheimer’s disease mouse models. Front. Pharmacol..

[142] Zboralski D., Hoehlig K., Eulberg D., Frömming A., Vater A. (2017). Increasing Tumor-Infiltrating T Cells through Inhibition of CXCL12 with NOX-A12 Synergizes with PD-1 Blockade. Cancer Immunol. Res..

[143] Giordano F.A., Layer J.P., Leonardelli S., Friker L.L., Turiello R., Corvino D., Zeyen T., Schaub C., Müller W., Sperk E. (2024). L-RNA Aptamer-Based CXCL12 Inhibition Combined with Radiotherapy in Newly-Diagnosed Glioblastoma: Dose Escalation of the Phase I/II GLORIA Trial. Nat. Commun..

